# The role of TRAF2 in pan-cancer revealed by integrating informatics and experimental validation

**DOI:** 10.3389/fphar.2025.1563435

**Published:** 2025-03-12

**Authors:** Xizheng Wang, Jianfeng Yuan, Chenchen Zhang, Lingyu Kong, Enzhen Wu, Jianxin Guo, Zhongbing Wu

**Affiliations:** ^1^ Arizona College of Technology, Hebei University of Technology, Tianjin, China; ^2^ College of Integrated Chinese and Western Medicine, Hebei Medical University, Shijiazhuang, China; ^3^ Affiliated Hospital of North China University of Science and Technology, Tangshan, Hebei, China

**Keywords:** pan-cancer analysis, tumor immune microenvironment, prognostic biomarker, hepatocellular carcinoma, targeted therapy

## Abstract

**Background:**

Tumor necrosis factor (TNF) receptor associated factor-2 (TRAF2) is an E3 ubiquitin ligase and scaffolding protein that contribute to the progression of various malignant tumors. However, the role of TRAF2 expression in epigenetic, cancer prognosis, and immune responses in tumor microenvironment is unclear.

**Methods:**

We used The Human Protein Atlas (HPA) database, TIMER 2.0 database, and TCGA database to evaluate TRAF2 expression in human normal and tumor tissues. Correlation of TRAF2 expression with mutations and epigenetic in tumors was evaluated using the cBioPortal platform and the GSCA database. To assess the prognostic value of TRAF2, we performed Kaplan-Meier plots and Cox regression analysis. LinkedOmics database was used for PANTHER Pathways enrichment analysis. The relationship between TRAF2 expression and immune checkpoint genes, as well as immune cell infiltration, was examined using TIMER 2.0 and the R language. Single-cell sequencing data and multiple immunofluorescence staining were used to observe the co-expression of TRAF2 on hepatocellular carcinoma cells and immune cells. Furthermore, using siRNA-mediated knockdown, we explored the potential role of TRAF2 in liver cancer cell biology.

**Results:**

Our findings indicate that TRAF2 is frequently mutated and significantly overexpressed in various types of cancers, and this overexpression is linked to a poor prognosis. The epigenetic alterations in TRAF2 was significant across various types of cancers. TRAF2 is associated with the levels of various immune checkpoint genes and multiple tumor-infiltrating immune cells, suggesting its potential involvement in tumor microenvironment. Of note, enrichment analysis revealed a significant correlation between TRAF2 and T cell activation, and single-cell sequencing indicated that TRAF2 was overexpressed in malignant cells and T cells. *In vivo* results demonstrated that TRAF2 was closely associated with T lymphocytes in hepatocellular carcinoma. The results of our *in vitro* experimental studies confirmed that the loss of TRAF2 function inhibits the malignant behavior of HepG2 cells in hepatocellular carcinoma.

**Conclusion:**

TRAF2 represents a potential prognostic biomarker and therapeutic target for cancer immunotherapy, particularly in patients with hepatocellular carcinoma.

## Graphical Abstract

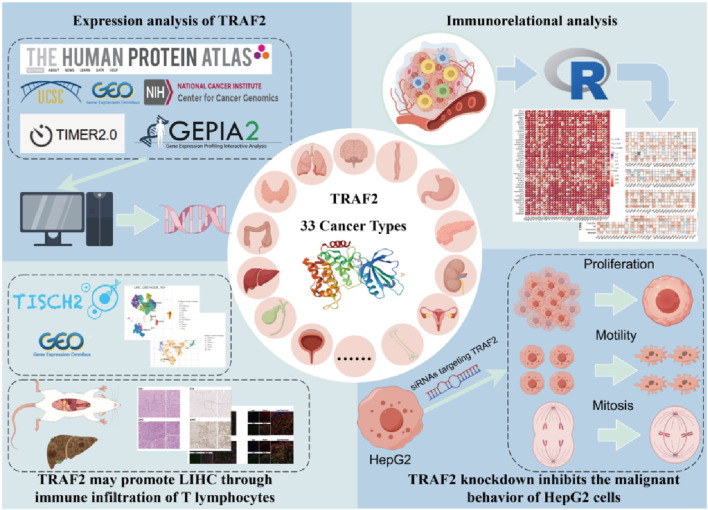

## 1 Introduction

Cancer is a complex disease that causes major public health problems worldwide. Data reported by the World Health Organization indicate that nearly 10 million deaths (almost one in six) in 2020 were caused by cancer (Sung et al., 2021). With the discovery of immune checkpoints (i.e., cytotoxic T lymphocyte antigen-4 (CTLA-4), V-domain immunoglobulin suppressor of T cell activation (VISTA), program cell-death 1 (PD-1), program cell-death ligand 1 (PD-L1) and T-cell immunoglobulin and mucin domain 3 (TIM-3)) (Deng et al., 2024; Mok et al., 2024; Zuo et al., 2024; Jin et al., 2024), immunotherapy, exemplified by immune checkpoint blockade (ICB), has revolutionized the treatment of cancer (Zhu et al., 2021). Related immune checkpoint genes play crucial roles in immune evasion and the response to immunotherapy in liver hepatocellular carcinoma (LIHC). Taking PD-1 as an example, CD8 effector/effector memory T (Teff/Tem) cells demonstrated a strong response to anti-PD-1 treatment (Guo et al., 2025; Matulich and Burger, 2025). In addition, anti-PD-L1/CTLA-4 bispecific antibodies demonstrated efficacy in the treatment of advanced unresectable or metastatic hepatocellular carcinoma in a clinical trial (Xu et al., 2025). However, some patients do not respond well to ICB therapy and exhibit a “cold tumor” phenotype (Han et al., 2024; Zhang et al., 2022; Zhang W. et al., 2024). Therefore, it is crucial to identify new biomarkers or immune-related therapeutic targets.

Tumor necrosis factor (TNF) receptor associated factor-2 (TRAF2) has a RING finger structure plus five zinc fingers (Wajant et al., 1999; Liu C. et al., 2024). TRAF2 recruits E2-Ub through its RING structural domain to mediate direct ubiquitin transfer to substrates, thereby conferring ubiquitination specificity (Pao et al., 2018). These modifications can have different effects on substrates. Therefore, TRAF2 is involved in a variety of cancer-related cellular processes, including activation of the NFκB signaling pathway (Wang Z. et al., 2024), stimulation of MAPKs signaling cascade transactivation (Shi and Sun, 2018), triggering ROS/endoplasmic reticulum (ER) stress signaling (Zhao et al., 2021), regulation of autophagy and apoptosis (Deen et al., 2024; Xu et al., 2023; Jiang et al., 2024), and control of cellular senescence (Yao et al., 2024). Several studies have found that TRAF2 is upregulated in various types of cancers, including hepatocellular carcinoma (Liang et al., 2023), esophageal squamous cell carcinoma (Zhang T. et al., 2024), lung squamous cell carcinoma (Nguyen et al., 2024), colorectal cancer (Teng et al., 2024), lymphoma (Vashisht et al., 2023), renal cell carcinoma (Ge et al., 2023), ovarian carcinoma (Zheng et al., 2022), and gastric carcinoma (Rossi et al., 2022). Several studies have demonstrated that TRAF2 is highly expressed in LIHC tissues (Yao et al., 2024; Liu Y. et al., 2024). Regarding the molecular mechanisms involved, it has been noted that the activation of the mTORC1 pathway can enhance the proliferation and survival of LIHC cells. Furthermore, TRAF2 can activate the mTORC1 pathway, thereby promoting the progression of LIHC (Liang et al., 2023; Li et al., 2024). A growing body of research has significantly demonstrated that TRAF2 plays a prominent role as an oncogene in cancer.

Previous studies were conducted on TRAF2 in some cancer types. However, these studies focused on a single type of cancer and lacked a pan-cancer perspective. The aim of this study was to evaluate the relationship between TRAF2 expression and the immune microenvironment by comprehensively analyzing TRAF2 expression and its prognostic value using bioinformatics. Moreover, our study illustrated the role of TRAF2 on the malignant behavior of hepatocellular carcinoma cells and the correlation between TRAF2 and immune infiltration of T lymphocytes in the tumor microenvironment. Our study underscores the prognostic importance of TRAF2 in pan-cancer and its potential as a cancer immunotherapy target.

## 2 Methods

### 2.1 Data collection

Gene expression RNAseq, somatic mutation (VarScan2 Variant Aggregation and Masking GDC Hub), and phenotype data (survival data GDC Hub) were obtained for 33 solid tumor types from the UCSC Xena database. Single-cell RNA-seq data were obtained from the GEO database, and the hepatocellular carcinoma GSE162616 dataset was included in the study. Supplementary Table 1 presents the complete nomenclature and corresponding abbreviations for the 33 cancer types examined in this study.

### 2.2 Expression analysis of TRAF2

The Human Protein Atlas (HPA) database (Sjöstedt et al., 2020) was utilized to examine the mRNA levels, as well as the protein localization and expression patterns of TRAF2 in healthy individuals. The involvement of TRAF2 in human diseases was elucidated through the OPENTARGET platform (Ochoa et al., 2023), which combines genetics and histology. The “Cancer Exploration” module in TIMER 2.0 (Li et al., 2020) was used to obtain TRAF2 gene expression differences between cancer and adjacent normal tissues. RNA-seq data were collected from the TCGA database for normal and tumor samples to examine differences in TRAF2 expression. For certain tumor types lacking normal samples in the TCGA database, we utilized the Analysis-Box Plot module in the GEPIA2 database (Tang et al., 2017) to acquire the relevant data for analysis and comparison. Meanwhile, the correlation between TRAF2 expression and tumor stage was obtained using the “Pathological Stage Plot” module of the GEPIA2 database.

### 2.3 TRAF2 mutation characterization and methylation analysis

To examine the mutational characteristics of TRAF2 in various cancers, we queried the site, type, and number of TRAF2 mutations in the “Mutation” module on the cBioPortal platform (De Bruijn et al., 2023). The mutation-protein topology of TRAF2 was obtained from the “Protein” module of the Protter database (Omasits et al., 2014). The “Mutation” module of the GSCA database (Liu et al., 2023) was utilized to examine the copy number variation (CNV) and methylation status of TRAF2, along with their correlation with mRNA expression levels. The XIANTAO platform was used to investigate the association between TRAF2 and the expression of methyltransferase genes. Lastly, the methylation level of TRAF2 in cancer was analyzed using the UALCAN database (Tang et al., 2023).

### 2.4 Survival analysis

Utilizing the one-way Cox analysis module available on the XIANTAO platform, we investigated the association between TRAF2 expression levels and three prognostic survival indicators in cancer patients. This analysis was conducted using data sourced from the TCGA database, encompassing overall survival (OS), disease-specific survival (DSS), and progression-free interval (PFI). Subsequent forest plots were generated to illustrate the hazard ratio (HR), 95% confidence interval, and p-value. An HR greater than 1 suggests that TRAF2 acts as a risk factor for cancer, while an HR less than 1 indicates a protective effect. Statistical significance was defined as p < 0.05.

### 2.5 Co-expressed genes and gene enrichment analysis

Through the LinkedOmics platform, cancer researchers can acquire, analyze, and compare multi-omics cancer data across various tumor types (Vasaikar et al., 2018). More specifically, the data utilized in this study was RNA-seq, and the statistical method employed was Pearson’s correlation test to identify which genes were co-expressed in the cancer targets. PANTHER Pathways enrichment analysis was then performed on TRAF2-related genes using Protein ANalysis THrough Evolutionary Relationships (PANTHER).

### 2.6 Immune cell infiltration analysis

Our study investigated the relationship between TRAF2 expression and tumor-infiltrating immune cells (TIICs) by utilizing the “immune-gene” module of TIMER 2.0. Our analysis primarily combined the QUANTISEQ, EPIC, MCPCOUNTER and TIMER algorithms. The results were presented as a heat map generated using the Spearman correlation test. In addition, we utilized the “ESTIMATE” package to compute the StromalScore, ImmuneScore, and ESTIMATEScore of the relevant samples. We then visualized them using the R packages “ggplot2,” “ggpubr,” “ggExtra.”

### 2.7 Immunotherapy correlation analysis

We used the R package “limma” to visualize the correlation between TRAF2 and related genes in a heatmap. Subsequently, we validated the correlation between TRAF2 and several immune checkpoint blockade-related genes, including PD-1, PD-L1, CTLA-4, LAG-3, CD47, and TIGIT, using the TIMER 2.0 database. To investigate the correlation between TRAF2 expression with tumor mutational burden (TMB), and microsatellite instability (MSI), the results were analyzed using Spearman’s correlation analysis and displayed as radar plots created with the “fmsb” package in R.

### 2.8 Single-cell sequencing analysis

We retrieved the single-cell expression patterns of immune cells in LIHC from the TISCH database to gain insight into the relevance of TRAF2 to immune cells in the tumor microenvironment. In addition, we obtained the single-cell RNA-seq dataset of LIHC (GSE162616) from the GEO database. We utilized the R package “Harmony” to integrate the de-batching process for GSE162616, conducted cellular annotation, visualized the downscaling using the UMAP function, and used Vlnplot method to visualize the details of TRAF2 expression.

### 2.9 Animal model establishment

Twelve male Wistar rats weighing 140–160 g were obtained from the Animal Center of Hebei Medical University. The Institutional Laboratory Animal Care Guidelines of the North China University of Science and Technology approved the study (SQ20230169). The rats were kept at a consistent temperature, exposed to a 12-h light–dark cycle. After 7 days of acclimatization, rats were divided into two groups. Control rats (Con, n = 6) were injected intraperitoneally with saline (vehicle for DEN) and olive oil (vehicle for CCl_4_). Model group (LIHC, n = 6) rats were injected intraperitoneally with 200 mg/kg of DEN and 0.5 mL/kg of CCl_4_ once a week for 3 weeks (Yakubu et al., 2020).

### 2.10 Hematoxylin and eosin (H&E), immunohistochemistry and immunofluorescence staining

All experimental rats were euthanized with sodium pentobarbital anesthesia (30 mg/kg, intraperitoneal injection), and then liver tissue was collected. Rat liver tissues were fixed in 4% paraformaldehyde for 24 h and subsequently embedded in paraffin. Following this, 4 μm paraffin sections were prepared, stained with hematoxylin and eosin (H&E), and examined microscopically. Anti-TRAF2 antibody was used for immunohistochemical detection of TRAF2 in paraffin-embedded rat liver samples (1:200, 26846-1-AP, Proteintech, United States). Positive staining from immunohistochemistry was subsequently quantified using Image-Pro Plus software, and the Integrated Optical Density (IOD) values were calculated.

Liver samples were deparaffinized and then antigenically repaired with Citric Acid Antigen Retrieval Solution (pH 6.0), after which the slides were blocked with 2% goat serum. Primary antibodies were then applied to the slides and left overnight at 4°C. The slides were washed three times with PBS (pH 7.4). Then, the secondary antibody was added and incubated for 50 min at room temperature in the dark. Primary antibodies were TRAF2 (1:100, 26846-1-AP, Proteintech, United States); CD45 (1:50, sc-1178, Santa Cruz); CD3 (1:50, sc-20047, Santa Cruz); CD4 (1:50, sc-19641, Santa Cruz); and CD8 (1:50, sc-1177, Santa Cruz). Slides were stained with DAPI for cell nuclei labeling and observed using a fluorescence microscope (Nikon Eclipse C1, Nikon, JPN).

### 2.11 Cell culture

The human liver hepatocellular carcinoma (LIHC) cell line HepG2 was obtained from the Wuhan Pricella Biotechnology (Wuhan, China). HepG2 Cells were cultured in HepG2 cell culture medium (Pricella, China). Cells were cultured in a 37°C, 5% CO_2_ humidified incubator (Thermo, United States).

### 2.12 Small interfering RNA (siRNA) transfection

Small interfering RNA (siRNA) targeting TRAF2 and non-targeting negative control (NC) were obtained from GenePharma (Shanghai, China) and transfected into the HepG2 cells using EL Transfection Reagent (TransGen, China) according to the manufacturer’s instructions. After 48 h of transfection, total mRNA as well as proteins were extracted to assess the transfection efficiency of siRNA. TRAF2 siRNA sequences are provided in Supplementary Table 2.

### 2.13 Real-time quantitative polymerase chain reaction (RT-qPCR)

Total RNA was isolated using the RNAiso Plus reagent (Takara, Japan) and subsequently reversely transcribed into cDNA with the SweScript All-in-One RT SuperMix for qPCR (G3337, Servicebio, China), following the manufacturer’s instructions. Quantitative real-time PCR (qRT-PCR) was performed using the SYBR Green method. Gene expression levels were quantified using the 2^−ΔΔCT^ method, with GAPDH as the internal reference gene. Detailed sequences of the specific primers used are provided in Supplementary Table 3.

### 2.14 Western blot (WB)

The transfected cells were lysed using RIPA lysis buffer (G2002, Servicebio, China), which contained protease and phosphatase inhibitors (G2006, G2007, Servicebio, China), on ice. The supernatant was subsequently collected. The enhanced bicinchoninic acid Protein Assay Kit (PC0020, Solarbio, China) was used to determine the quality of the total protein collected. Protein samples were mixed with sampling buffer (RW0301, ReportBio, China) and denatured by heating at 95°C for 10 min. The samples were then loaded onto a 10% SDS-PAGE gel for electrophoresis and transferred to a PVDF membrane, which was subsequently sealed. The PVDF membrane was incubated with TRAF2 Polyclonal Antibody (1:1,000, 26846-1-AP, Proteintech, United States) and β-Actin Rabbit Monoclonal Antibody (1:50,000, AC026, ABclonal, United States) for 2 h at room temperature. Following this, the membrane was incubated with goat anti-rabbit IgG (H+L) secondary antibody (1:5,000, S1002, Ripatec, China) at 4°C overnight. Protein expression was detected using an ECL kit (HY-K1005, MCE, United States) and analyzed with ImageJ software.

### 2.15 Cell counting kit-8 (CCK-8) assay

After transfection, cells were seeded into 96-well plates at a density of 2 × 10^3^ cells per well. Following 24 and 48 h of incubation, 10 μL of CCK-8 solution was added to each well, and the plates were incubated for an additional 2 h. The optical density (OD) was measured at 450 nm using a microplate reader (Bio-Rad Laboratories, Hercules, United States). The formula was calculated as follows:

$$cell\;viability=\frac{{OD}_{experimental\;group}-{OD}_{blank\;group}}{{OD}_{control\;group}-{OD}_{blank\;group}}\times 100\%$$

### 2.16 Assessment of cell proliferation by EdU assay

After the transfected cells were inoculated into 6-well plates and cultured for 48 h, they were incubated with an EdU working solution (10 μM, C0075S, Beyotime Institute of Biotechnology, China) for 2 h at 37°C. It was fixed with 4% paraformaldehyde for 10 min at room temperature, incubated with DAPI (10 μg/mL, BL105A, Biosharp, China) for 5 min at room temperature, and then observed and photographed using a fluorescence microscope (Nikon Eclipse C1, Nikon, JPN) before being analyzed with ImageJ software.

### 2.17 Determination of lactic acid content

Lactate production was detected using a lactate assay kit (A019-2-1, Nanjing Jiancheng Bioengineering Institute, China) according to the manufacturer’s protocol. Briefly, the transfected cells were cultured in a 6-well plate. After 48 h of culture, the cells were collected by centrifugation. Subsequently, 300 μL of double-distilled water was added, and the mixture was placed in an ice-water bath to create a cell suspension. Afterward, the cell suspension (20 μL) was mixed with the reaction mixture (containing 1 mL of enzyme working solution and 20 μL of color developer) for 10 min at 37°C in a water bath, followed by the addition of 1 mL of termination solution. A microplate reader (Bio-Rad Laboratories, Hercules, United States) was used to measure absorbance at 530 nm.

### 2.18 Cell cycle evaluation

The transfected cells were cultured in 6-well plates. After 48 h of incubation, the cells were collected and resuspended. We added propidium iodide (PI, CCS012, MultiSciences Biotech Co., China) and permeabilization solution, and incubated the mixture for 30 min at 25°C in the dark. The cells were collected, and the cell cycle was analyzed using a flow cytometer (Attune NxT, Thermo Fisher Scientific Inc., United States).

### 2.19 Scratch assay

The transfected cells were cultured in six-well plates. When the cells reached 80%–90% confluence, a scratch was created using a 1,000-μL pipette tip and then washed with PBS to remove any suspended cells. Images were captured at 24 h and 48 h, and the healing area of the scratches was measured using ImageJ.

### 2.20 Invasion assays

The Transwell system was utilized to evaluate the invasiveness of tumor cells. Both the transfected and control cells were inoculated at a density of 2.5 × 10^4^ cells per well in the upper chamber of the Transwell, while medium containing bovine serum was added to the lower chamber. The cells were cultured in a cell culture incubator for 24 and 48 h. After incubation, the cells in the upper chamber were removed, fixed with 4% paraformaldehyde, and the membrane was wiped with a cotton swab. The cells were then stained with crystal violet and photographed.

### 2.21 Statistics analysis

Statistical analyses were conducted using R, version 4.3.3, and GraphPad Prism 8. The Student’s t-test was used to analyze the statistical difference. Differences were defined as significance at p < 0.05.

## 3 Results

### 3.1 TRAF2 expression in normal human tissues

**FIGURE 1:**
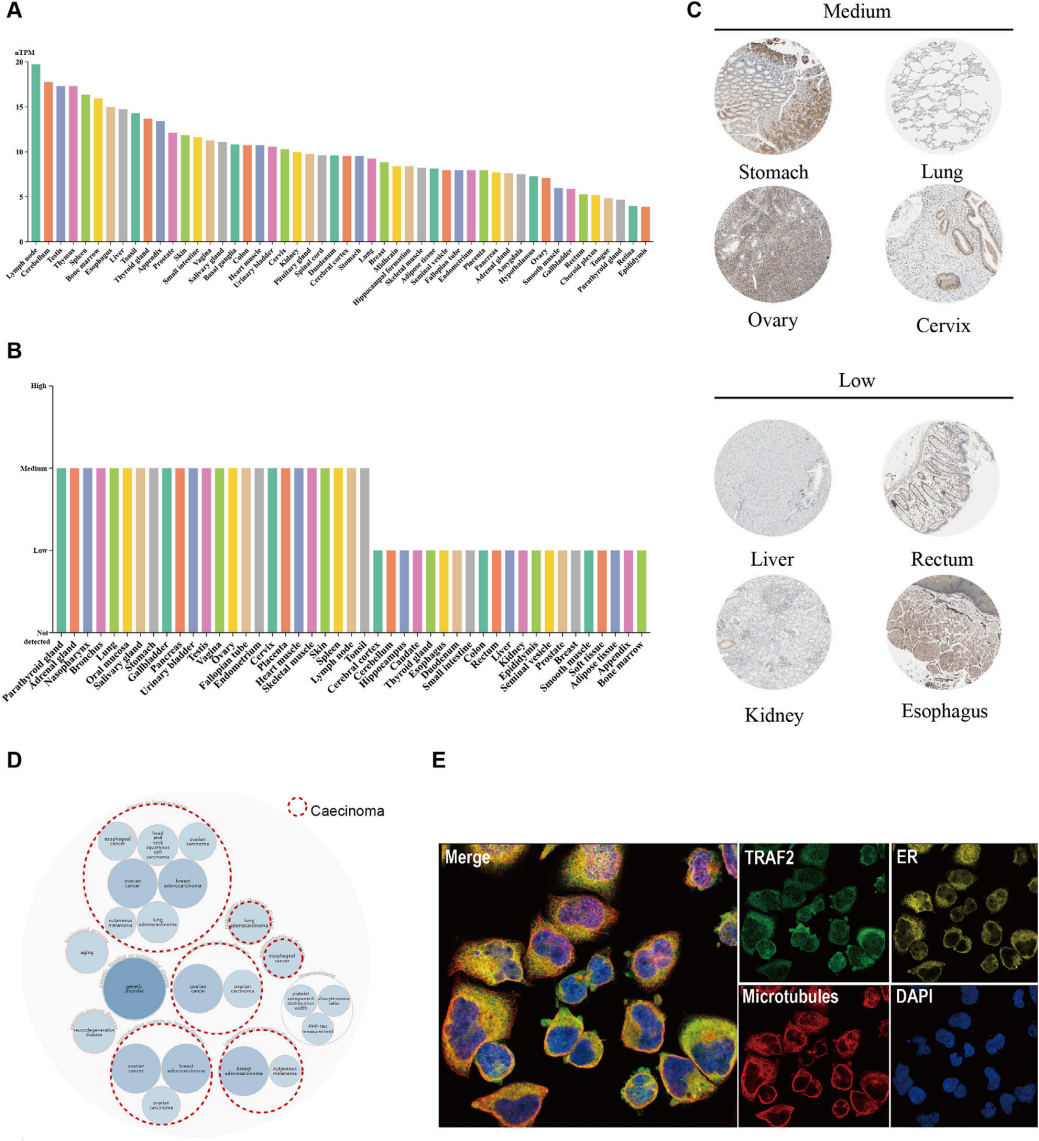
TRAF2 expression in various normal human tissues and its association with diseases. **(A)** mRNA expression profile of TRAF2 in normal human tissues. **(B)** Protein expression data of TRAF2 in normal human tissues. **(C)** Representative IHC images of TRAF2 expression in normal stomach, lung, ovary, cervix, liver, kidney, rectum, and esophagus. **(D)** Disease network map of associated diseases of TRAF2, with red circular dotted lines indicating an association with cancer. **(E)** Subcellular localization profile of TRAF2 in human A-431 cells.

To observe the expression of TRAF2 in normal human tissues, we used the HPA database to evaluate the mRNA levels, protein expression localization, and TRAF2 levels. The tissues with the highest TRAF2 mRNA levels were lymph node, cerebellum, testis, thymus and spleen (Figure 1A). As shown in Figure 1B, we examined the expression of TRAF2 at the protein level and observed that it was expressed at medium and low levels in various tissues, showing significant differences. The expression of TRAF2 in the cytosol was also observed in the immunohistochemical results, which showed representative tissue staining results of different expression levels (Figure 1C). The levels of expression varied across different tissues: stomach (medium), lung (medium), ovary (medium), cervix (medium), liver (low), kidney (low), rectum (low), and esophagus (low). We integrated genetics and histology for gene-disease network interaction analysis and demonstrated that TRAF2 is implicated in the progression of diseases in multiple domains, including cancer, hereditary diseases, aging, and neurodegenerative disorders (Figure 1D). TRAF2 is located in the cytosol, suggesting that it may have a signaling function (Figure 1E).

### 3.2 TRAF2 expression in various tumor tissues

**FIGURE 2:**
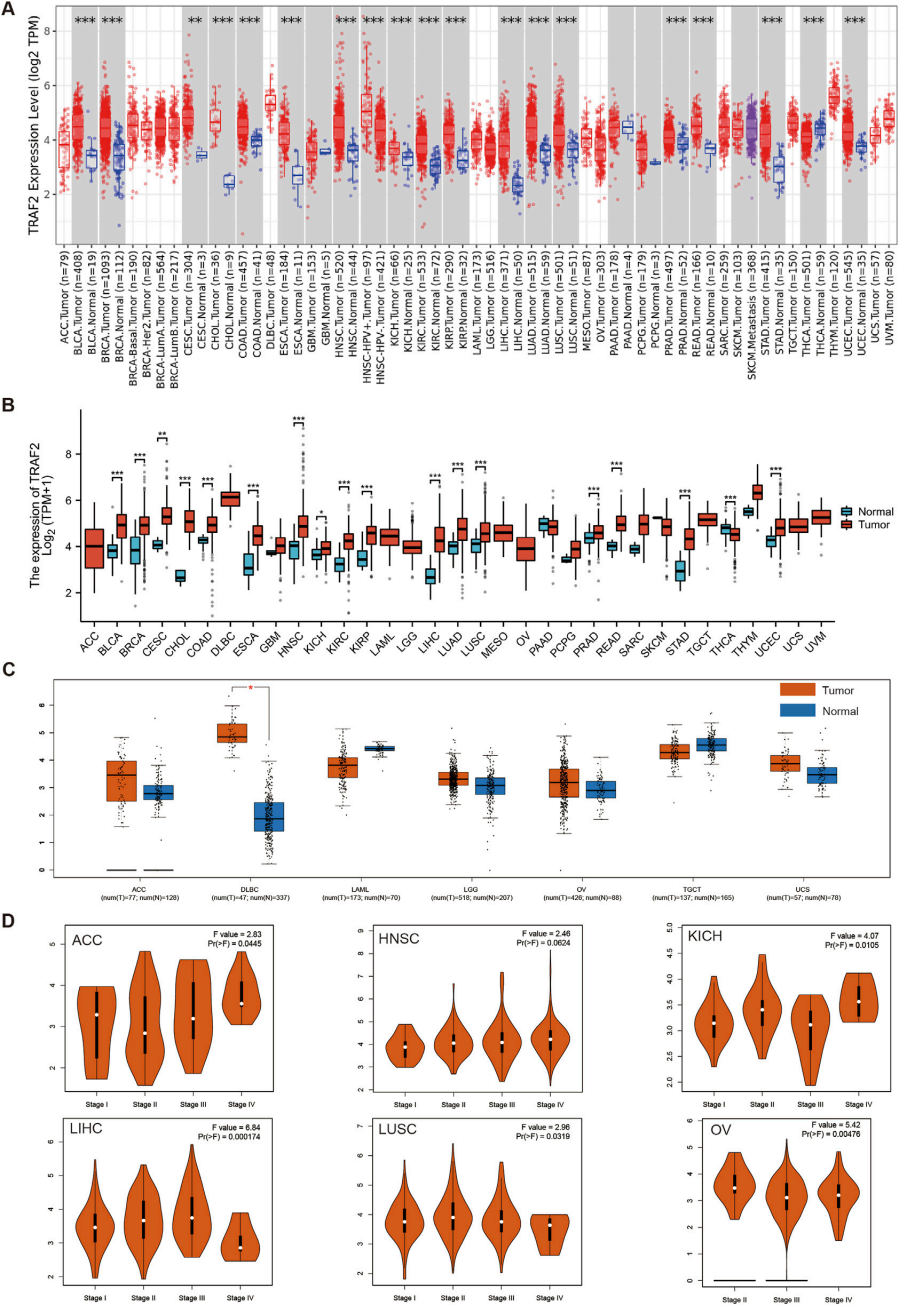
TRAF2 expression in various tumor tissues. **(A)** TIMER2.0 database analysis of TRAF2 expression differences in 33 human tumors and their corresponding adjacent tissues. **(B)** TCGA in conjunction with the GTEx database to analyze differences in TRAF2 expression between tumor and normal tissues in pan-cancer. **(C)** Complementary analysis of TRAF2 expression differences between ACC, DLBC, LAML, LGG, MESO, OV, TGCT, UCS, UVM, and normal tissues by GEPIA2 platform. **(D)** The GEPIA2 platform was used to analyze the correlation between TRAF2 expression and cancer clinical stage (*p < 0.05, **p < 0.01, ***p < 0.001).

We analyzed the differential expression of TRAF2 in 33 human tumors and their corresponding adjacent tissues. We used both the TIMER 2.0 database and the TCGA database in conjunction with the GTEx database for this analysis. In which, TRAF2 mRNA expression was increased in BLCA, BRCA, CESC, CHOL, COAD, ESCA, HNSC, KICH, KIRC, KIRP, LIHC, LUAD, LUSC, PRAD, READ, STAD, UCEC, and decreased in THCA (Figures 2A, B). To enhance the credibility of the results, the GEPIA2 platform was applied to the cancer types with missing normal samples in the above two analysis methods (for example, ACC, DLBC, LAML, LGG, MESO, OV, TGCT, UCS, UVM). The mRNA expression of TRAF2 was increased in DLBC. However, data on normal samples for MESO and UVM were still missing (Figure 2C). In addition, we applied the GEPIA2 database to investigate the expression of TRAF2 in different cancer stages. The expression of TRAF2 was found to be associated with the clinicopathological stages of ACC, HNSC, KICH, LIHC, LUSC, and OV (Figure 2D). Notably, the most significant correlation was observed with the clinical stage of LIHC (F value = 6.84, Pr (>F) = 0.000174). Supplementary Figure 1 displays the results with low correlation.

### 3.3 TRAF2 mutation and methylation in tumors

**FIGURE 3:**
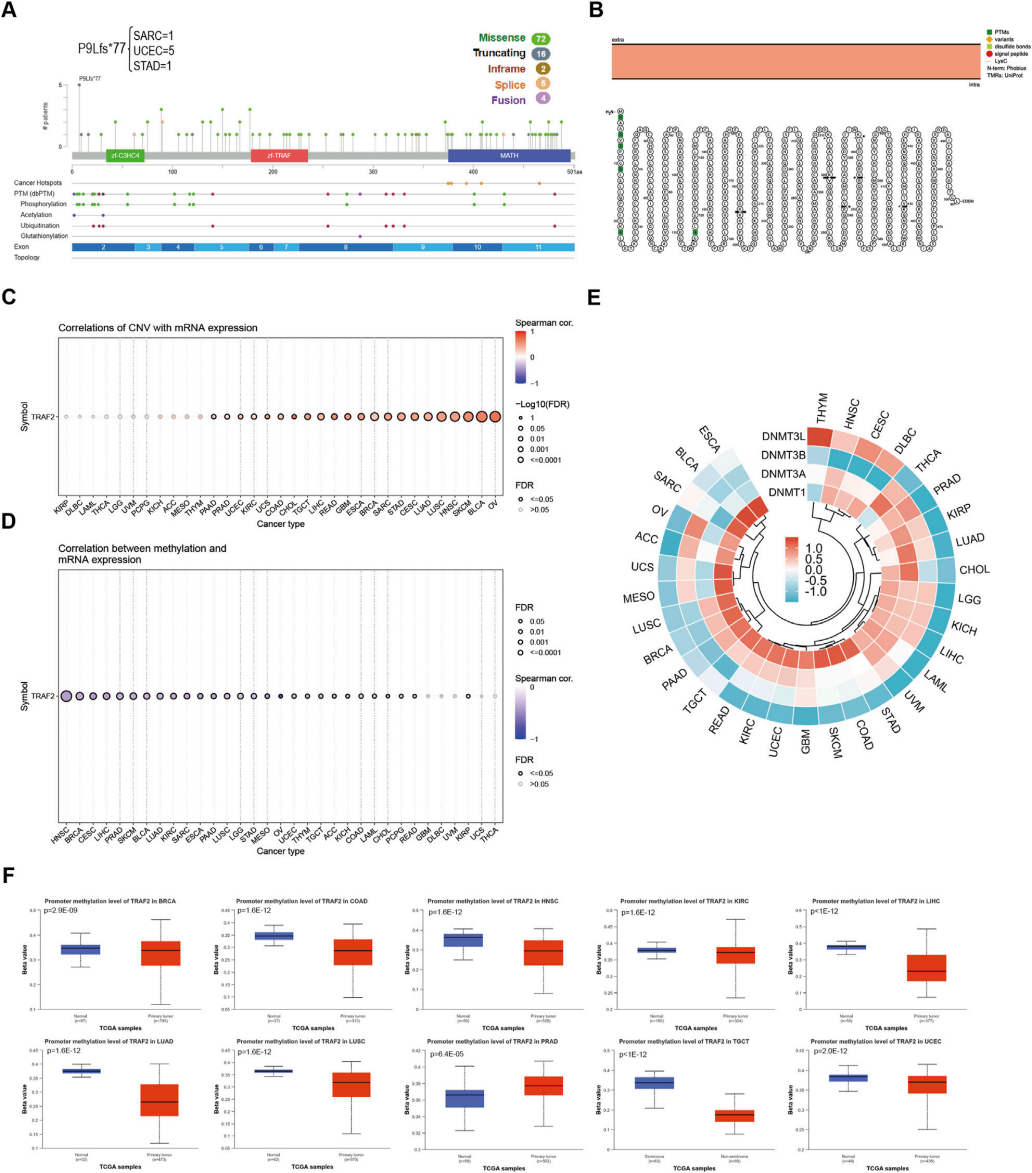
Aberrant mRNA expression of TRAF2 is associated with mutations and methylation modifications. **(A)** Mutation analysis of TRAF2 in the CBioPortal database. **(B)** TRAF2 gene mutation-protein topology. **(C)** Correlation of CNV and TRAF2 mRNA expression in the GSCA dataset. **(D)** Correlation of methylation and TRAF2 mRNA expression in the GSCA dataset. **(E)** Correlation of TRAF2 mRNA with four methyltransferases (DNMT1, DNMT3A, DNMT3B, and DNMT3L). **(F)** Analysis of TRAF2 methylation status using the UALCAN dataset.

We used the cBioPortal platform for the mutation analysis of TRAF2. The results showed that a total of 99 mutation sites were identified in the 501 amino acids of TRAF2, including 72 missense mutations, 16 truncating mutations, two inframe mutations, five splice site mutations, and four fusion mutations. Among these mutations, P9Lfs*77 was the most common mutation site (Figure 3A). TRAF2 gene mutation-protein topology revealed multiple protein translational modifications (PTMs) (Figure 3B). To further explore the mechanism of aberrant TRAF2 mRNA expression, we analyzed the gene expression in relation to gene copy number variation (CNV) and methylation. The results of the GSCA database showed that TRAF2 expression in OV, BLCA, SKCM, HNSC, LUSC, CESC, STAD, SARC, BRCA, ESCA, GBM, READ, LIHC, TGCT, CHOL, COAD, UCS, KIRC, UCEC, PRAD, and PAAD patients had a significant positive CNV correlation (Figure 3C). Methylation is a common form in PTMs, and the results indicated that methylation levels were correlated with mRNA expression in all 28 tumors except THCA, UCS, UVM, DLBC, and GBM, with the strongest correlations in HNSC, BRCA, CESC, LIHC, and PTAD (Figure 3D). To further explore the reasons for the inconsistent methylation levels across tumors, we analyzed the correlation between TRAF2 and the four methyltransferase genes (DNMT1, DNMT3A, DNMT3B, and DNMT3L). The results suggested a significant correlation between these genes and TRAF2 in KIRP, LGG, KICH, LIHC, UVM, and GBM (Figure 3E). By analyzing the promoter methylation status of TRAF2 in cancers, it was observed that the methylation level decreased in 9 cancers and increased in 1 cancer (Figure 3F). The results of sample sizes <30, and those showing no significant difference, are presented in Supplementary Figure 2.

### 3.4 Correlation between TRAF2 expression levels and cancer prognosis

**FIGURE 4:**
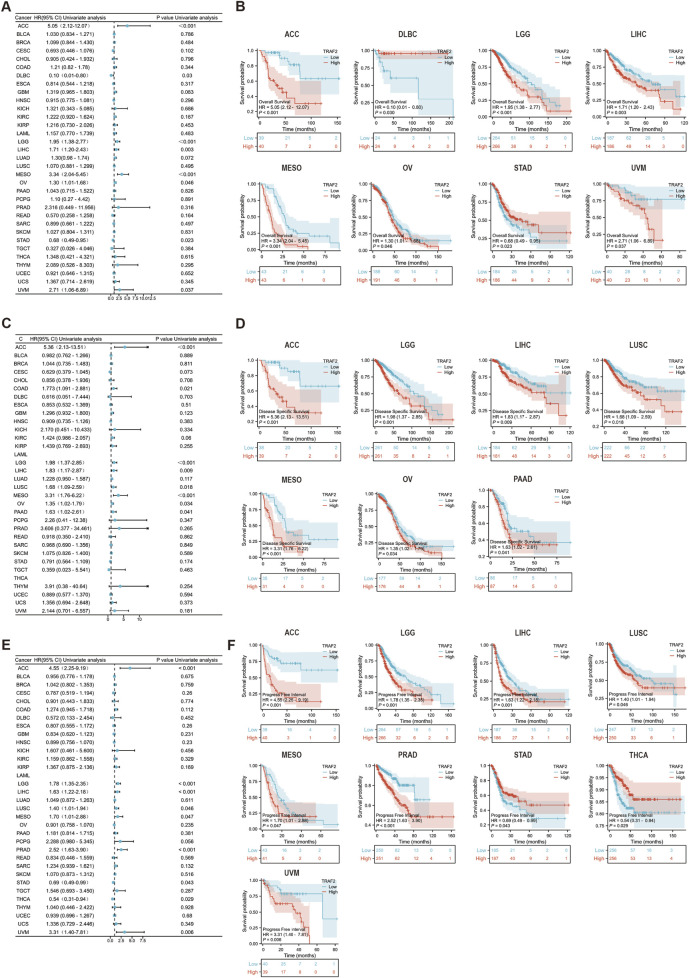
High expression of TRAF2 is positively associated with a poor prognosis in cancer patients. **(A, C, E)** Correlation of TRAF2 expression with OS, DSS, and PFI in patients with various cancer types. **(B)** Kaplan-Meier analysis of OS including ACC, LGG, LIHC, MESO, OV, UVM, DLBC, and STAD. **(D)** Kaplan-Meier analysis of DSS, including ACC, LGG, LIHC, LUSC, MESO, OV, and PAAD. **(F)** Kaplan-Meier analysis of PFI, including ACC, LGG, LIHC, LUSC, MESO, PRAD, UVM, STAD, and THCA.

We explored the relationship between TRAF2 expression and OS, DSS, and PFI in 33 cancer types by TCGA RNA-seq and clinical data. As shown in Figures 4A, B, the analysis of OS revealed that patients with high TRAF2 expression in ACC, LGG, LIHC, MESO, OV, and UVM had a poor prognosis. Conversely, high TRAF2 expression in DLBC and STAD indicated a favorable prognosis. In ACC, LGG, LIHC, LUSC, MESO, OV, and PAAD, high expression of TRAF2 is associated with poor DSS (Figures 4C, D). Using PFI as a criterion, high TRAF2 expression in ACC, LGG, LIHC, LUSC, MESO, PRAD, and UVM indicated poor prognosis, in contrast to high TRAF2 expression levels that were positively correlated with a favorable prognosis in STAD and THCA (Figures 4E, F). Overall, OS, DSS, and PFI were significantly lower in hepatocellular carcinoma patients with high TRAF2 expression (hazard ratio [HR] > 1.5, p < 0.001). This association was most pronounced across multiple cancer types.

### 3.5 Gene enrichment analysis of TRAF2 in cancer

**FIGURE 5:**
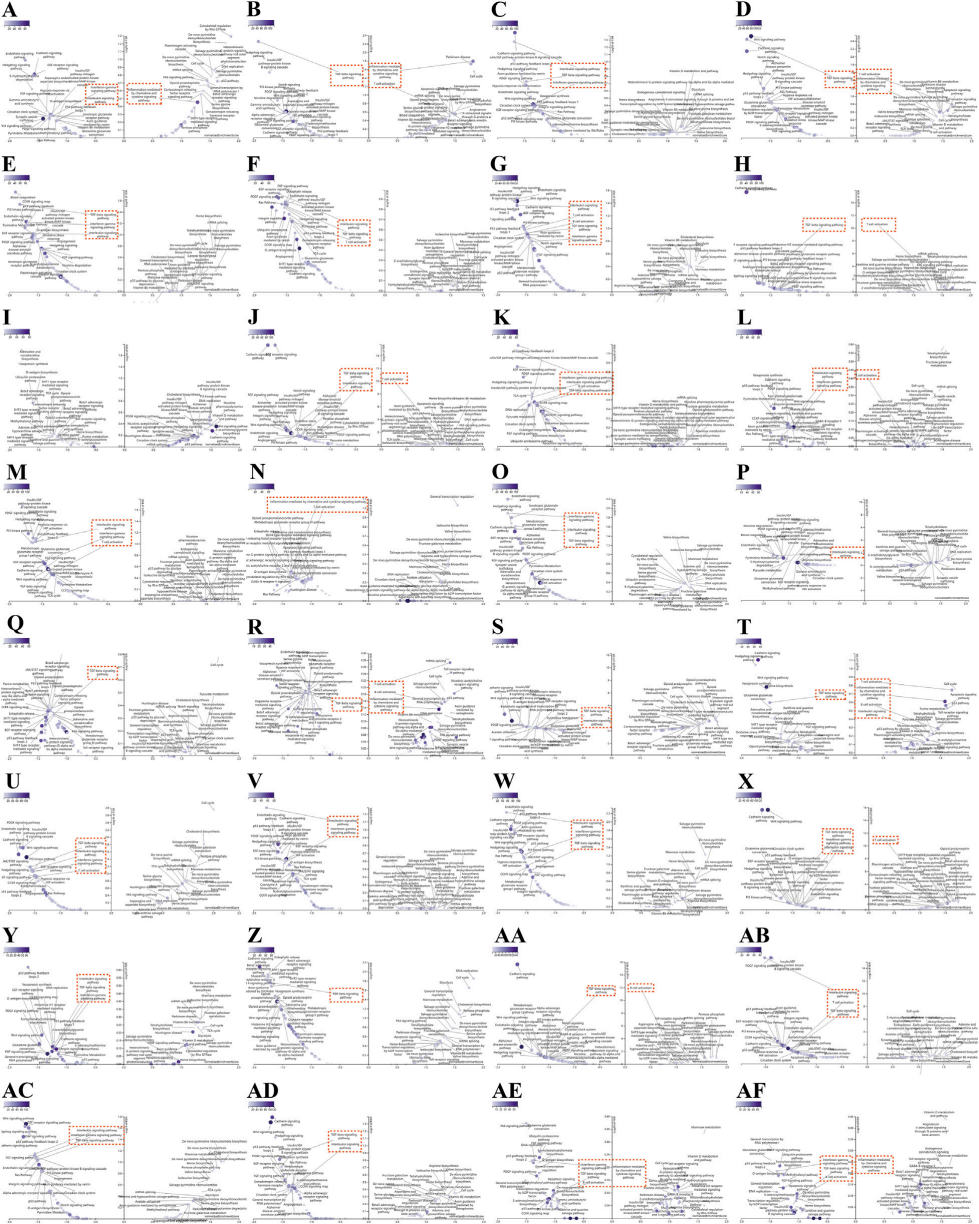
Association of immune-related pathways and TRAF2 expression in 32 cancers with GSEA-PANTHER functional enrichment. TRAF2 in **(A)** ACC, **(B)** BLCA, **(C)** BRCA, **(D)** CESC, **(E)** CHOL, **(F)** COAD, **(G)** DLBC, **(H)** ESCA, **(I)** GBM, **(J)** HNSC, **(K)** KICH, **(L)** KIRC, **(M)** KIRP, **(N)** LAML, **(O)** LGG, **(P)** LIHC, **(Q)** LUAD, **(R)** LUSC, **(S)** MESO, **(T)** OV, **(U)** PAAD, **(V)** PCPG, **(W)** PRAD, **(X)** SARC, **(Y)** SKCM, **(Z)** STAD, **(AA)** TGCT, **(AB)** THCA, **(AC)** THYM, **(AD)** UCEC, **(AE)** UCS, **(AF)** UVM PANTHER functional enrichment pathway. Those marked in red are immune-related pathways (FDR ≤ 0.05). The left side indicates negative enrichment, and the right side indicates positive enrichment.

We conduct more in-depth studies on the biological functions of TRAF2 in various types of cancers. For this purpose, we analyzed the co-expression of genes associated with TRAF2 in the LinkedOmics database for 32 types of cancers and used heatmaps to display the top 50 genes that were positively or negatively correlated with TRAF2 (Supplementary Figure 3). After that, we performed PANTHER Pathways set enrichment analysis of TRAF2 using the GSEA tool in the LinkedOmics database in the form of volcano diagrams. Notably, we found that immune-related pathways were enriched in 31 types of cancers, except for GBM and READ (no relevant data in the LinkedOmics database) (Figure 5). Specifically, we observed that high TRAF2 expression was mainly negatively correlated with immune-related pathways, such as T cell activation, B cell activation, TGF -β signaling pathway, Interleukin signaling pathway, Interferon -γ signaling pathway, and inflammation mediated by chemokine and cytokine signaling pathway. These findings suggest a potential correlation between high TRAF2 expression and immunosuppression in tumor microenvironment (TME).

### 3.6 Correlation between TRAF2 expression and immune cell infiltration

**FIGURE 6:**
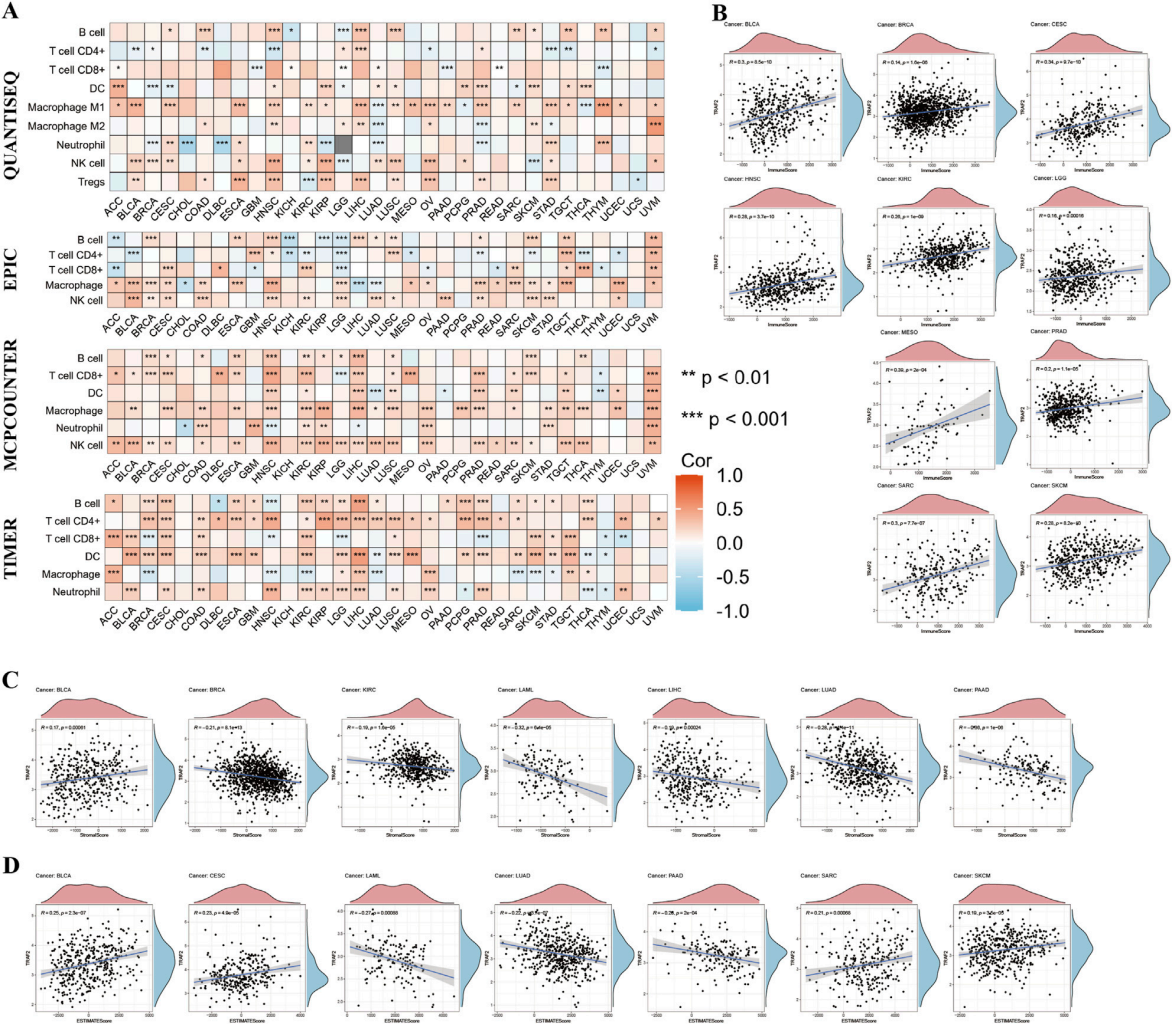
The Relationship between TRAF2 and immune cell infiltration. **(A)** The relationship between TRAF2 and varying levels of immune cell infiltration was analyzed using the MCPCOUNTER, QUANTISEQ, EPIC, and TIMER tools. **(B–D)** Cancer types for which TRAF2 expression was significantly associated with ImmuneScore, StromalScore, and ESTIMATEScore. *p < 0.05, **p < 0.01, ***p < 0.001.

The above findings suggest that TRAF2 is primarily associated with immune cell infiltration in TME. We further analyzed the association between TRAF2 expression levels and the extent of tumor-infiltrating immune cell infiltration in TME using the TIMER2.0 database (Supplementary Figure 4). The results demonstrated by four analytical methods, MCPCOUNTER, QUANTISEQ, EPIC, and TIMER, show that TRAF2 is positively correlated with the level of infiltration of DC cells, NK cells, and Macrophages. In contrast, TRAF2 expression was negatively correlated with B cells, CD4^+^ T cells, and CD8^+^ T cells (Figure 6A). Further investigation of the pan-cancer relationship between TRAF2 expression and TME revealed that TRAF2 expression was significantly correlated with ImmuneScore, StromalScore, and ESTIMATEScore in 14 different types of cancers, namely BLCA, BRCA, CESC, HNSC, KIRC, LAML, LGG, LIHC, LUAD, MESO, PAAD, PRAD, SARC, and SKCM. According to our results, the three tumor types with the strongest correlation between TRAF2 expression and ImmuneScore were MESO, CESC, and BLCA (Figure 6B). The three cancer types with the strongest correlation between TRAF2 and StromalScore were PAAD, LAML, and LUAD (Figure 6C). The three cancer types with the highest correlation between TRAF2 and ESTIMATEScore were PAAD, LAML, and BLCA (Figure 6D).

### 3.7 Association of TRAF2 expression with immune checkpoints and immunotherapy

**FIGURE 7:**
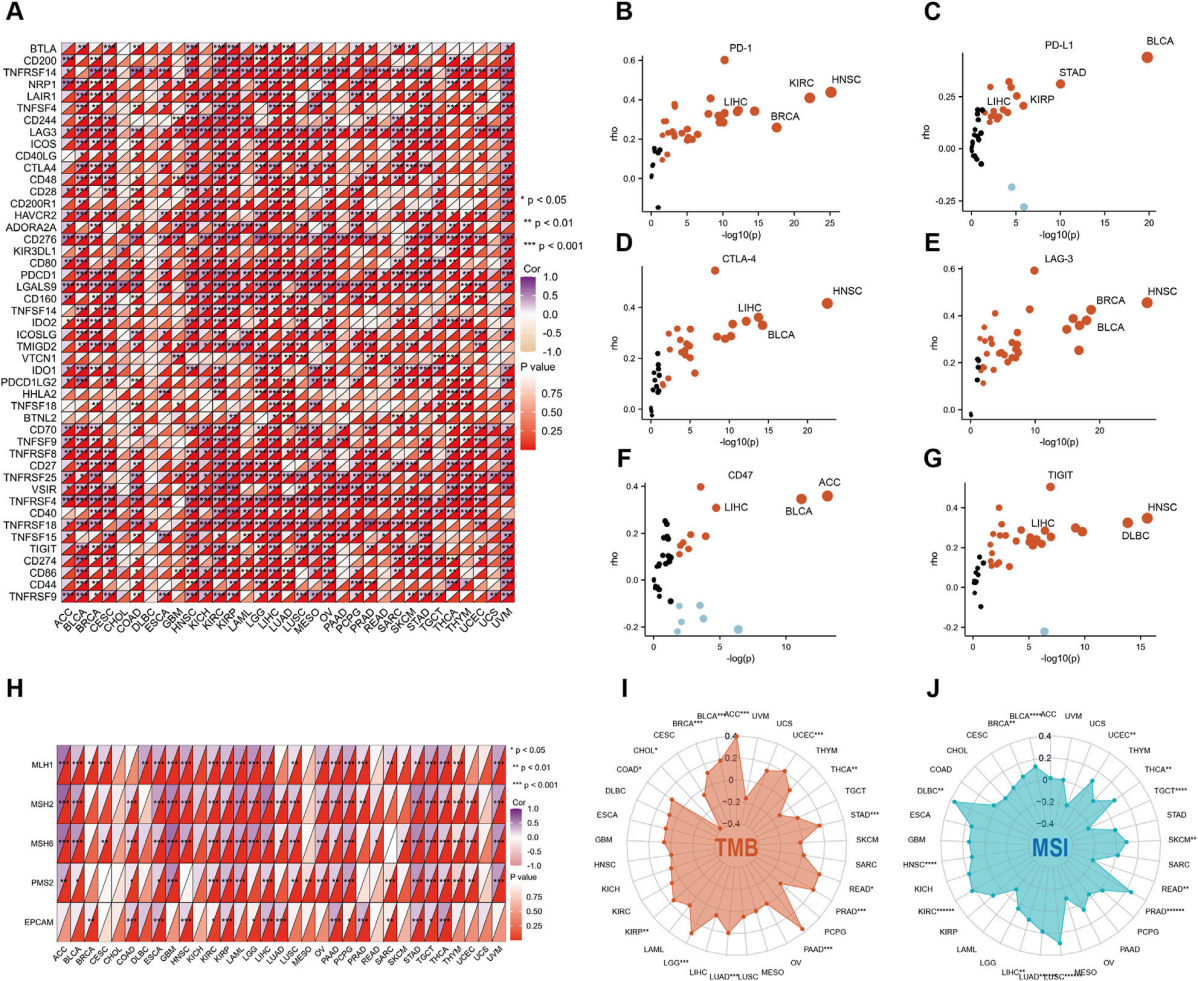
TRAF2 is associated with immune checkpoints and immunotherapy. **(A)** Correlation of TRAF2 with 47 immune checkpoint genes. **(B–G)** Correlation of TRAF2 with PD-1, PD-L1, CTLA-4, LAG-3, CD47, and TIGIT. **(H)** Correlation of TRAF2 with MMR-related genes including MLH1, MLH2, MLH6, PMS2 and EPCAM. **(I)** Correlation of TRAF2 expression with TMB in pan-cancer. **(J)** Correlation of TRAF2 expression with MSI in pan-cancer. *p < 0.05, **p < 0.01, ***p < 0.001.

Considering the strong correlation of TRAF2 with immune cells, we explored the association of TRAF2 with immunomodulatory molecules. Overall, TRAF2 was shown to positively correlate with the expression of genes associated with immune checkpoints in most cancer types (Figure 7A), which supports the hypothesis that TRAF2 may be effective in cancer immunotherapy. We verified the correlation between TRAF2 and several immune checkpoint blocking genes (including PD1, PD-L1, CTLA-4, LAG-3, CD47, and TIGIT) in the TIMER 2.0 database, and the results were consistent with those of previous studies. The most significant positive correlation between TRAF2 and these genes was observed in HNSC, BLCA, BRCA, LIHC (Figures 7B–G).

We further explored the correlation between TRAF2 and dynamic immune-related features, including mismatch repair (MMR), tumor mutation burden (TMB), and microsatellite instability (MSI). Significant correlations were found between TRAF2 and several MMR-associated genes in ESCA, KIRP, LIHC, STAD, and THCA, such as MutL homolog 1 (MLH1), MutS homolog 2 (MSH2), MutS homolog 6 (MSH6), Homolog 2 (PMS2), and Epithelial Cell Adhesion Molecule (EPCAM) (Figure 7H). We found that TRAF2 was positively correlated with ACC, BLCA, BRCA, COAD, KIRP, LGG, LUAD, PAAD, PRAD, READ, STAD, and TMB of UCEC, but negatively correlated with CHOL and THCA (Figure 7I). TRAF2 was positively correlated with MSI in BLCA, BRCA, DLBC, HNSC, KIRC, LIHC, LUAD, LUSC, PRAD, SKCM, THCA, and UCEC, but negatively correlated with MSI in READ and TGCT (Figure 7J).

### 3.8 Correlation of TRAF2 with immune cells in the hepatocellular carcinoma tumor microenvironment

**FIGURE 8:**
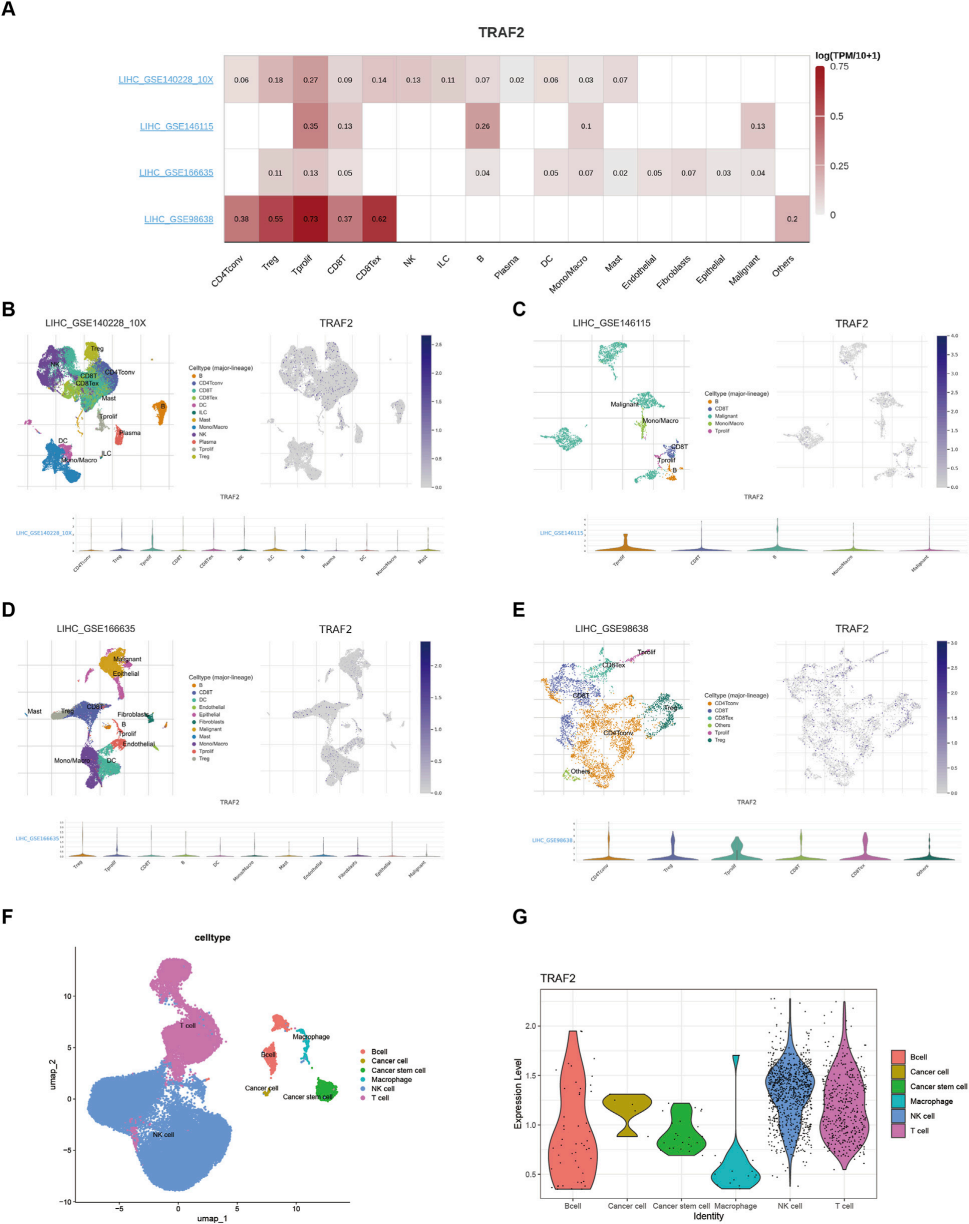
Single-cell analysis suggests that TRAF2 is closely associated with T cells in the LIHC. **(A)** Heat map of TRAF2 correlation with immune cells. **(B–E)** Annotation of all cell types and percentage and expression of TRAF2 in GSE140228,GSE146115,GSE166635,and GSE98638. **(F, G)** Cell type annotation with TRAF2 expression in validation set GSE162616.

Since significant correlations between TRAF2 expression and both prognosis and immune cell infiltration were observed in LIHC, we explored TRAF2 expression in immune cells using the TISCH database in LIHC. The scRNA-seq data retrieved from the TISCH database for the GSE140228,GSE146115,GSE166635,and GSE98638 cohorts showed that TRAF2 was expressed predominantly in T cells (Figures 8A–E). We observed that TRAF2 showed higher expression levels in T cells and tumor cells, which is consistent with the results in the enrichment analysis. In addition, we validated this finding using the LIHC scRNA-seq dataset obtained from the GEO dataset (GSE162616), and the results suggested the presence of high expression of TRAF2 in T-cells, NK-cells, and tumor cells (Figures 8F, G).

### 3.9 TRAF2 promotes LIHC progression through infiltration of T lymphocytes

**FIGURE 9:**
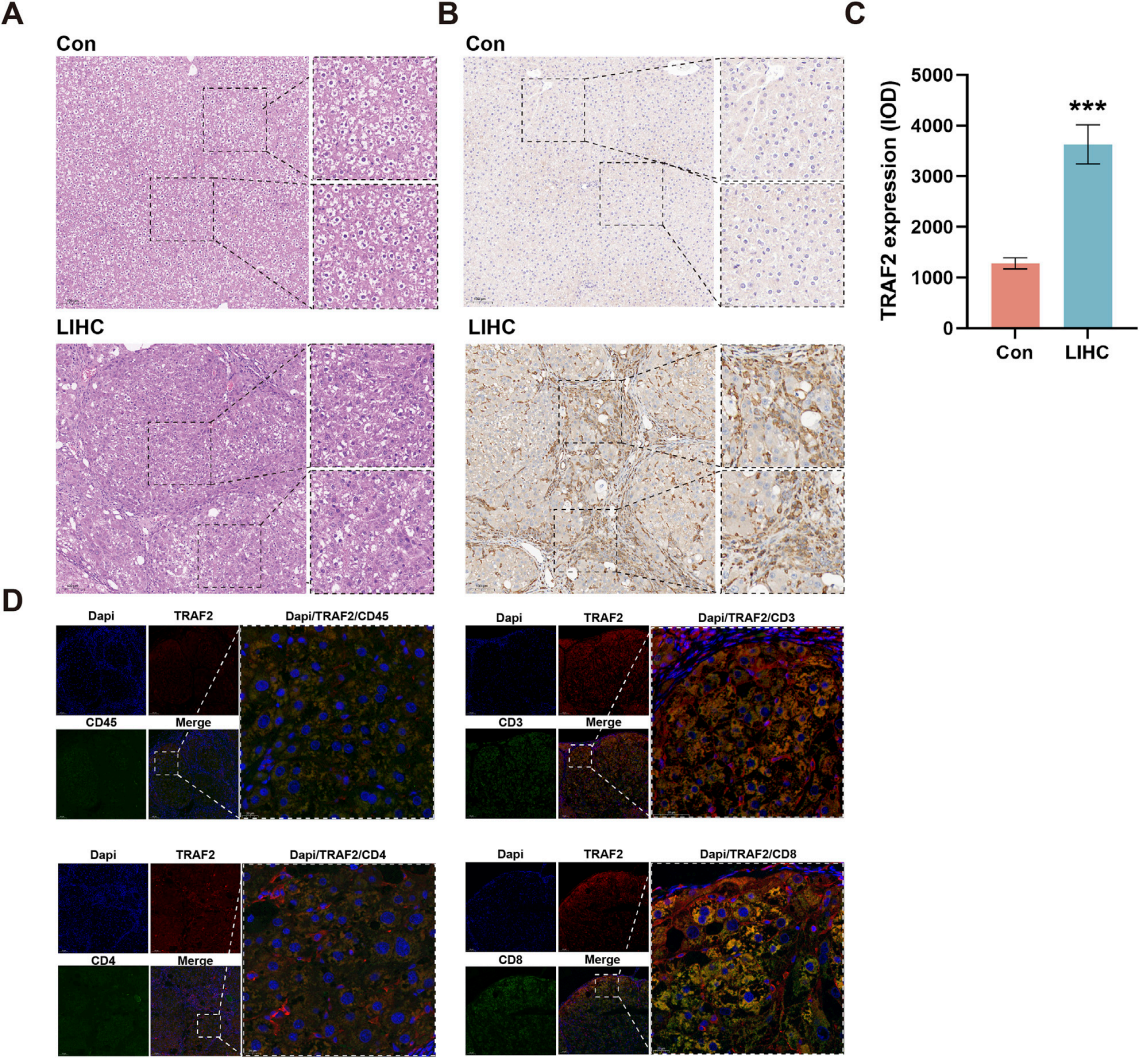
Expression of TRAF2 in liver tissue of LIHC rats, and correlation between TRAF2 and T lymphocytes. **(A)** Hematoxylin and eosin examination of histopathological changes. **(B)** TRAF2 staining of rat liver tissue, brown for positive staining. **(C)** The statistical results of immunohistochemistry were utilized to compare the IOD values between the two groups. **(D)** Immunofluorescence analysis of TRAF2 localization at the cellular level in liver tissues. Representative double staining immunofluorescence images show that LIHC tissues express CD45 (green), CD3 (green), CD4 (green), CD8 (green), with TRAF2 (red). Dapi (blue) is used for nuclear staining. Observed under 10× and 40× microscopes. ***p < 0.001.

We established a DEN- and CCl4-induced LIHC rat model to study the relationship between TRAF2 and T lymphocytes. Firstly, the histopathology of the rat liver was evaluated. The liver lobules of rats in the HCC group were structurally disorganized, with an increased size of cell nucleus. Some of the nucleus showed binucleated or multinucleated characteristics, and there was an increased presence of fibrous tissue in the interstitial space of the liver (Figure 9A). Immunohistochemical analysis showed a significant increase in TRAF2 in LIHC tissues compared to the Con group (Figures 9B, C). In addition, immunofluorescence co-localization was used to identify the correlation of TRAF2 expression with total T cells, CD3^+^ T cells, CD4^+^ T cells, and CD8^+^ T cells in liver tissues of rats in the LIHC group. TRAF2 positive staining overlapped with CD45, CD3, CD4, and CD8 (Figure 9D). The results suggest that TRAF2 is important for T-lymphocyte-dominated immunomodulation in the LIHC tumor microenvironment and may influence LIHC development through it.

### 3.10 TRAF2 knockdown inhibits the malignant behavior of HepG2 cells

**FIGURE 10:**
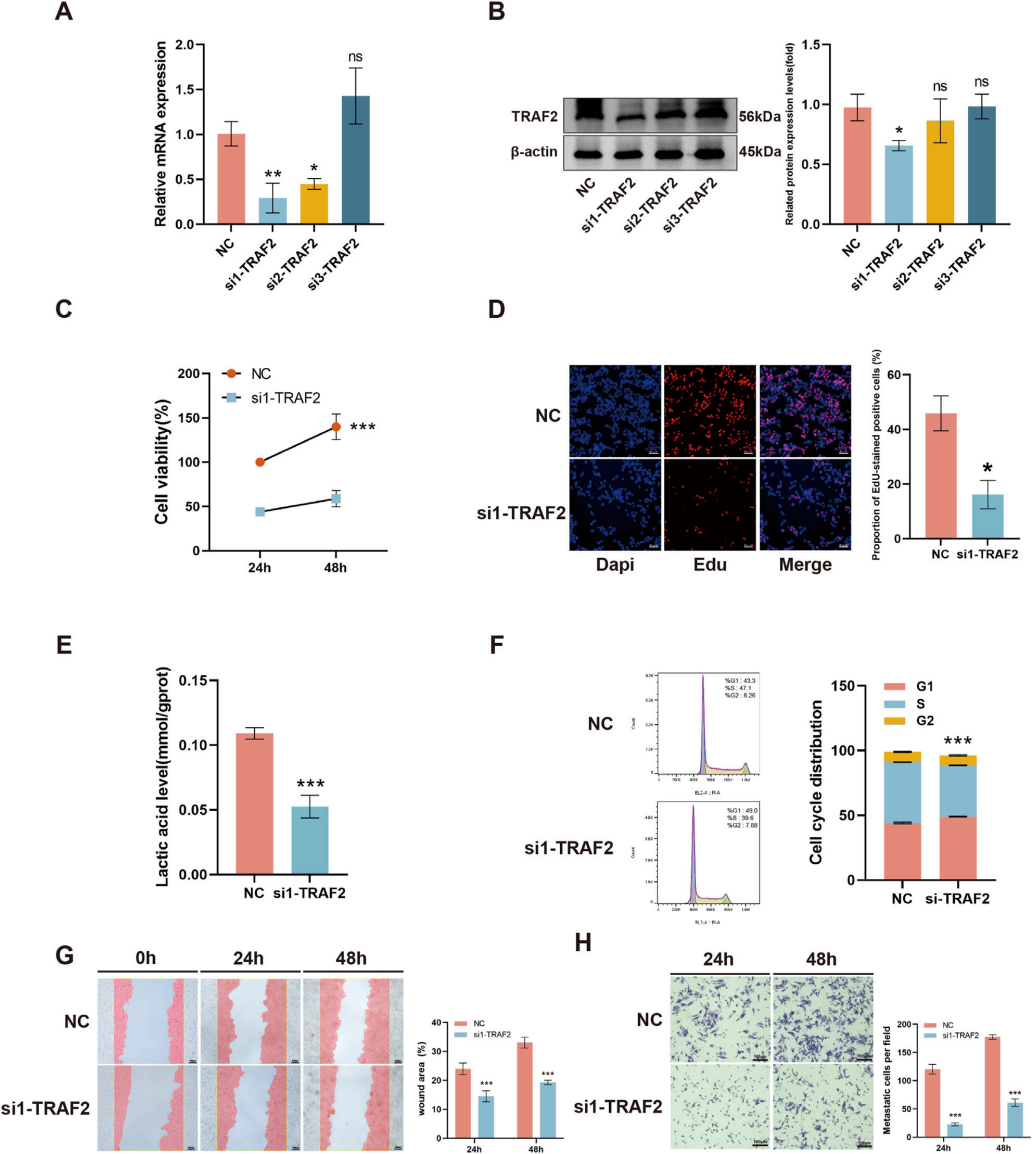
Effect of knockdown of TRAF2 on malignant behavior of HepG2 cells. **(A, B)** Knockdown efficiency of TRAF2 mRNA levels versus protein levels in HepG2 cells. **(C, D)** Effect of knockdown of TRAF2 on the proliferative capacity of HepG2 cells. **(E)** Effect of knockdown of TRAF2 on lactate content in HepG2 cells. **(F)** Effect of knockdown of TRAF2 in HepG2 cell cycle. **(G, H)** Effect of knockdown of TRAF2 and migration and invasion ability of HepG2 cells. *p < 0.05, **p < 0.01, ***p < 0.001.

We transfected HepG2 cells with three different siRNAs targeting TRAF2. The qRT-PCR with Western blot results showed that si1-TRAF2 had a higher knockdown efficiency (Figures 10A, B). We selected si1-TRAF2 for subsequent experiments. CCK-8 with EdU fluorescent labeling assay showed that TRAF2 knockdown inhibited HepG2 cell proliferation (Figures 10C, D). In addition, the lactate content assay showed that TRAF2 knockdown reduced the lactate content in HepG2 cells, indicating a reduced proliferative capacity (Figure 10E). Flow cytometry was used to examine the changes in cell cycle progression after knockdown of TRAF2, which exhibited a higher G1 phase cell cycle arrest compared to control cells (Figure 10F). In addition, the effect of knocking down TRAF2 on the migration and invasion ability of HepG2 cells was observed, and the results showed that the migration and invasion ability of HepG2 cells in the TRAF2 knockdown group was significantly reduced compared with that of the control group (Figures 10G, H).

## 4 Discussion

As a key player in the tumor necrosis factor receptor (TNFR) signaling pathway, TRAF2 serves a dual regulatory role in both innate and adaptive immunity (So, 2022). This study thoroughly analyzed the significant association between TRAF2 and the development of 33 different cancers (Chang et al., 2024; Wang M. et al., 2024; Wu et al., 2024). It also explored the relationship between TRAF2 expression levels and various factors, including mutations, epigenetic alterations, prognosis, immune cell infiltration, immune checkpoint molecules, and immune cells. This comprehensive approach offers a multidimensional perspective for cancer research.

We found that TRAF2 mRNA and protein are expressed at medium and low levels in normal human tissues. TRAF2 is widely distributed in most cancer tissues, and its expression level is much higher than that in normal tissues. The expression level of TRAF2 correlates with the clinicopathological stage of ACC, HNSC, KICH, LIHC, LUSC, and OV. Mutations and aberrant DNA methylation are known to cause dysregulation of gene expression in cancer (Nishiyama and Nakanishi, 2021), and our findings are consistent with previous studies (Hill et al., 2020; Moon et al., 2021), indicating TRAF2 has a high rate of gene mutations. In addition, TRAF2 showed altered methylation levels in 28 tumor promoter regions and significant correlations with four methyltransferase genes. The widespread upregulation of TRAF2 suggests its potential significance in a wide range of cancer types, and through precise analysis of clinical prognostic data, we found that TRAF2 is a factor for poor prognosis in a wide range of cancers. These results suggest that TRAF2 may play a crucial role in the development of various types of cancers and could be used as a biomarker for early cancer detection and follow-up.

Currently, the molecular mechanisms of TRAF2 are mainly being investigated in specific cancer types, and its role in pan-cancer is unclear. Previous studies have shown that TRAF2, as an articulating protein with E3 ligase activity, mediates interactions with plasma membrane receptors, kinases, phosphatases, other E3 ligases, and deubiquitinases to regulate a variety of signaling pathways, including NFκB, MAPK, tumor necrosis factor receptor-associated death domain protein (TRADD), proto-oncogene cJun (JUN), Wnt/β-catenin, and other signaling pathways (Siegmund et al., 2022). Notably, pathway enrichment analysis based on GSEA-PANTHER in pan-cancer showed that high TRAF2 expression was negatively correlated with the enrichment of immune cell related pathways (such as, T cell activation, B cell activation, and inflammation mediated by chemokine and cytokine signaling pathways).

It has been shown that TME consists of cellular and non-cellular elements surrounding the tumor and significantly affects the biological behavior of tumor cells as well as affects treatment responsiveness (Tang et al., 2024; Vilela et al., 2024; Zhou et al., 2024). We examined the effect of TRAF2 on the tumor immune microenvironment by assessing immune cell infiltration using four different analytical methods (MCPCOUNTER, QUANTISEQ, EPIC, and TIMER) using the ESTIMATE algorithm to analyze the ImmuneScore, the StromalScore, and the ESTIMATEScore. It was revealed that the infiltrating abundance of tumor-infiltrating immune cells (TIIC) was significantly correlated with TRAF2 expression in a wide range of malignancies, and that TRAF2 expression was strongly associated with CD4T cells, CD8T cells, and B cells. Notably, TRAF2 binding motifs are required for proper B cell CD40 function (Lu et al., 2023). Experimental evidence indicates that TRAF2 deficiency increases T cell sensitivity to IL-15 and supports the survival and function of effector CD8^+^ T cells (Villanueva et al., 2015). In contrast, TRAF2 overexpression facilitates the differentiation of Th17 cells by activating STAT3 signaling (Nagashima et al., 2018). This process may contribute to tumor angiogenesis and the establishment of an immune-suppressive microenvironment through the secretion of IL-17. Together, these mechanisms contribute to the suppression of effector T-cell function within the TME, resulting in a phenotype. Interestingly, the absence of TRAF2 greatly enhances their sensitivity of tumor cells to immunotherapy (Vredevoogd et al., 2019). In addition, TRAF2 synergistically regulates the TGF-β pathway alongside Smurf2 (Li et al., 2024). Furthermore, TGF-β blockers have been demonstrated to reverse the inhibition of NK cell function (Sparano et al., 2025). Therefore, co-targeting TRAF2 in conjunction with immune checkpoints or metabolic pathways may represent a novel strategy to enhance the efficacy of immunotherapy in hepatocellular carcinoma.

Immune-related gene expression is considered a predictive marker for immunotherapy in several cancers (Zhong et al., 2023). We analyzed the correlation of TRAF2 with 47 immune checkpoint genes, and the data showed a strong positive correlation between TRAF2 and these immune checkpoint genes, such as HNSC, BLCA, BRCA, and LIHC. MMR, TMB, and MSI promote immunogenicity and improve the response to immune checkpoint inhibitors. They are independent predictive immunotherapy biomarkers (Palova et al., 2024; Samstein et al., 2019). Our analyses showed a significant correlation between TRAF2 expression and TMB and MSI status, suggesting a complex, interactive relationship between TRAF2 and tumor immune microenvironment, indicating that TRAF2 can regulate anti-tumor immune responses.

The results indicated that the tumor immune microenvironment of LIHC is highly complex. Furthermore, the elevated expression of TRAF2 in LIHC was significantly correlated with poor patient prognosis, suggesting that it plays a crucial role in the development of LIHC. Further, we explored the details of malignancies and immune cell expression in LIHC through multiple single-cell datasets, examining TRAF2 expression in the tumor immune microenvironment (TIME). We found that TRAF2 is highly expressed in tumor cells and T cells (CD8Tex, Tprolif, Treg, CD8T, and CD4T cells). Additionally, we constructed a rat model of LIHC and confirmed the aberrant expression of TRAF2 in LIHC using IHC experiments. We also verified TRAF2 in CD3^+^, CD4^+^, and CD8^+^ T cells using multiplexed immunofluorescence staining. These findings suggest that TRAF2 may promote LIHC progression through T cells. TRAF2 has been reported to be involved in T cell activation processes (Wu et al., 2021), generation of memory T cells (Jaeger-Ruckstuhl et al., 2020), maintenance of effector and memory CD8 T cells (Xie et al., 2021), naive T cell activation (Jaeger-Ruckstuhl et al., 2024) and M2 macrophage polarization (Xu et al., 2023). Specifically, TRAF2 facilitates the ubiquitin-mediated degradation of NIK proteins in T cells by recruiting the E3 ubiquitin ligases cIAP1 and cIAP2. This process inhibits nonclassical NF-κB signaling, ultimately leading to the suppression of T cell activation. In addition, TRAF2 binds to the CD27 receptor, inhibiting the differentiation of naïve T cells into effector T cells while promoting the formation of memory T cells through the TRAF2-SHP1 axis. This mechanism may explain the observed correlation between high TRAF2 expression and reduced CD8^+^ T cell infiltration in the current study.

Our findings confirm that TRAF2 may play an immunomodulatory role by affecting T cells. In addition, TRAF2 interacts with Smad ubiquitination regulatory factor 2 (Smurf2), which precisely regulates the TGF-β pathway (Li et al., 2024; Fu et al., 2020). Furthermore, TGF-β specifically regulates the differentiation and function of trNK cells (Sparano et al., 2025).

A part of the study showed that activation of the mTORC1 pathway by TRAF2 promotes the proliferation and survival of hepatocellular carcinoma (Liang et al., 2023). In addition, the invasiveness of hepatocellular carcinoma is affected by the TRAF2-regulated WNT/PI3K/NF-kb signaling pathway (Wang Z. et al., 2024). HepG2 cell line is a widely used and well-characterized model in LIHC research. Its biological properties are relatively stable, making it suitable for a large number of related studies, including investigations into cell proliferation (Amelimojarad et al., 2025), cell migration (Wang et al., 2025; Zhao et al., 2024), lipid metabolism (Jia et al., 2016), the cell cycle (Addy et al., 2025), and autophagy (Wen et al., 2025), among other aspects. We knocked down TRAF2 in hepatocellular carcinoma HepG2 cells, and silencing TRAF2 resulted in a significant reduction in cell proliferation, intracellular lactate content, cell cycle, and migratory and invasive ability. These findings support the pro-tumorigenic role of TRAF2 in LIHC.

Although this study systematically reveals the role of TRAF2 in various cancers, limitations remain. Firstly, some of our results are limited to a single method or database and lack cross-validation of data from multiple sources. To address this limitation, we have selected multiple datasets for validation to mitigate the drawbacks of relying on a single database. In addition, although these findings point to new directions for follow-up studies, there is still a need to further investigate the potential biological function and molecular mechanisms of TRAF2 in tumor immunity using experiments.

## 5 Conclusion

In conclusion, TRAF2 is significantly overexpressed in 17 different cancer types and is strongly associated with poor prognosis. In addition, TRAF2 was found to be strongly associated with immune cell infiltration in tumor microenvironment and efficacy of immunotherapy, with particular association with T cells, which was experimentally validated in hepatocellular carcinoma. These findings provide new candidates for future cancer drug therapy, immunotherapy targets, and prognostic biomarkers.

## Data Availability

Publicly available datasets were analyzed in this study. This data can be found here: https://www.ncbi.nlm.nih.gov/geo/query/acc.cgi?acc=GSE162616,GEO, GSE162616.
