# Supplementary material for: The role of TRAF2 in pan-cancer revealed by integrating informatics and experimental validation
Source: Front Pharmacol. 2025 Mar 12;16:1563435. doi: 10.3389/fphar.2025.1563435 (PMC11937082; doi:10.3389/fphar.2025.1563435)
Supplement: Supplementary file 1 [file DataSheet1.pdf]

Supplemental material

Table 1. Full names and abbreviations of the 33 cancer types in TCGA.

Serial number	Cancer Types	Abbreviations
1	Adrenocortical carcinoma	ACC
2	Bladder Urothelial Carcinoma	BLCA
3	Breast invasive carcinoma	BRCA
4	Cervical squamous cell carcinoma and endocervical adenocarcinoma	CESC
5	Cholangiocarcinoma	CHOL
6	Colon adenocarcinoma	COAD
7	Lymphoid Neoplasm Diffuse Large B-cell Lymphoma	DLBC
8	Esophageal carcinoma	ESCA
9	Glioblastoma multiforme	GBM
10	Head and Neck squamous cell carcinoma	HNSC
11	Kidney Chromophobe	KICH
12	Kidney renal clear cell carcinoma	KIRC
13	Kidney renal papillary cell carcinoma	KIRP
14	Acute Myeloid Leukemia	LAML
15	Brain Lower Grade Glioma	LGG
16	Liver hepatocellular carcinoma	LIHC
17	Lung adenocarcinoma	LUAD
18	Lung squamous cell carcinoma	LUSC
19	Mesothelioma	MESO
20	Ovarian serous cystadenocarcinoma	OV
21	Pancreatic adenocarcinoma	PAAD
22	Pheochromocytoma and Paraganglioma	PCPG
23	Prostate adenocarcinoma	PRAD
24	Rectum adenocarcinoma	READ
25	Sarcoma	SARC
26	Skin Cutaneous Melanoma	SKCM
27	Stomach adenocarcinoma	STAD
28	Testicular Germ Cell Tumors	TGCT
29	Thyroid carcinoma	THCA
30	Thymoma	THYM
31	Uterine Corpus Endometrial Carcinoma	UCEC
32	Uterine Carcinosarcoma	UCS
33	Uveal Melanoma	UVM

Table 2 Website addresses used in this study.

Database name	Web site of the database
GEO	https://www.ncbi.nlm.nih.gov/geo/

UCSC Xena	https://xena.ucsc.edu/
HPA	https://www.proteinatlas.org
OPENTARGET	https://platform.opentargets.org/
TIMER 2.0	http://timer.cistrome.org/
TCGA	http://cancergenome.nih.gov
GEPIA2	http://gepia2.cancer-pku.cn/#index
GTEx	https://www.gtexportal.org/home/
cBioPortal	https://www.cbioportal.org/
Protter	http://wlab.ethz.ch/protter/start/
GSCA	https://guolab.wchscu.cn/GSCA/#/
XIANTAO	https://www.xiantaozi.com/about
UALCAN	https://ualcan.path.uab.edu/index.html
LinkedOmics	www.linkedomics.org/login.php
TISCH	http://tisch.comp-genomics.org/home/

Table 3. The sequence of siTRAF2.

	sense	antisense
NC	5'-UUC UCC GAA CGU GUC ACG UTT-3	5'-ACG UGA CAC GUU CGG AGA ATT-3
si1-TRAF2	5'-CUG AAA GAA UAC GAG AGC UTT-3	5'-AGC UCU CGU AUU CUU UCA GTT-3
si2-TRAF2	5'-GCU CAU GCU GAC CGA AUG UTT-3	5'-ACA UUC GGU CAG CAU GAG CTT-3
si3-TRAF2	5'-GUC AAG ACU UGU GGC AAG UTT-3	5'-ACU UGC CAC AAG UCU UGA CTT-3

Table 4. The sequence of the TRAF2 primer and GAPDH primer

	sequence(5'-3')
TRAF2-FORWARD	CACAGGGCTGACATGCAAAC
TRAF2-REVERSE	CGTGGGCTCCTTCCTCAAAT
GAPDH-FORWARD	GTCTCCTCTGACTTCAACAGCG
GAPDH-REVERSE	ACCACCCTGTTGCTGTAGCCAA

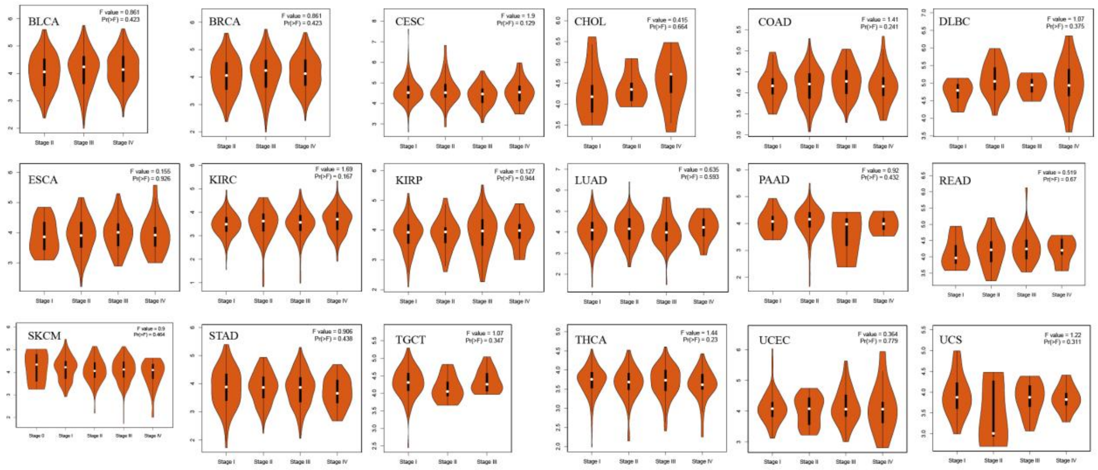

Fig.1 The GEPIA2 platform analyzed the correlation between TRAF2 expression and cancer pathological stage.

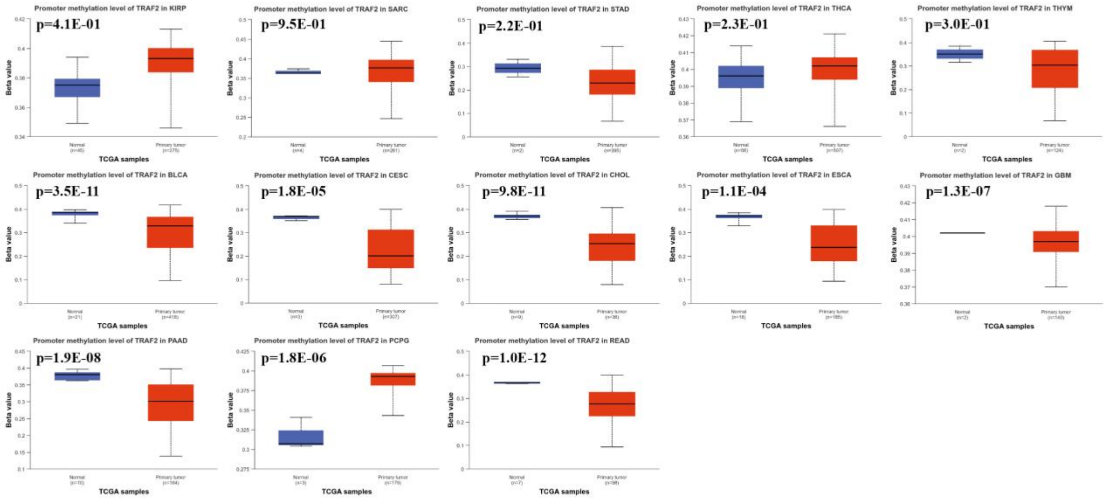

Fig.2 UALCAN database analysis of TRAF2 methylation status.

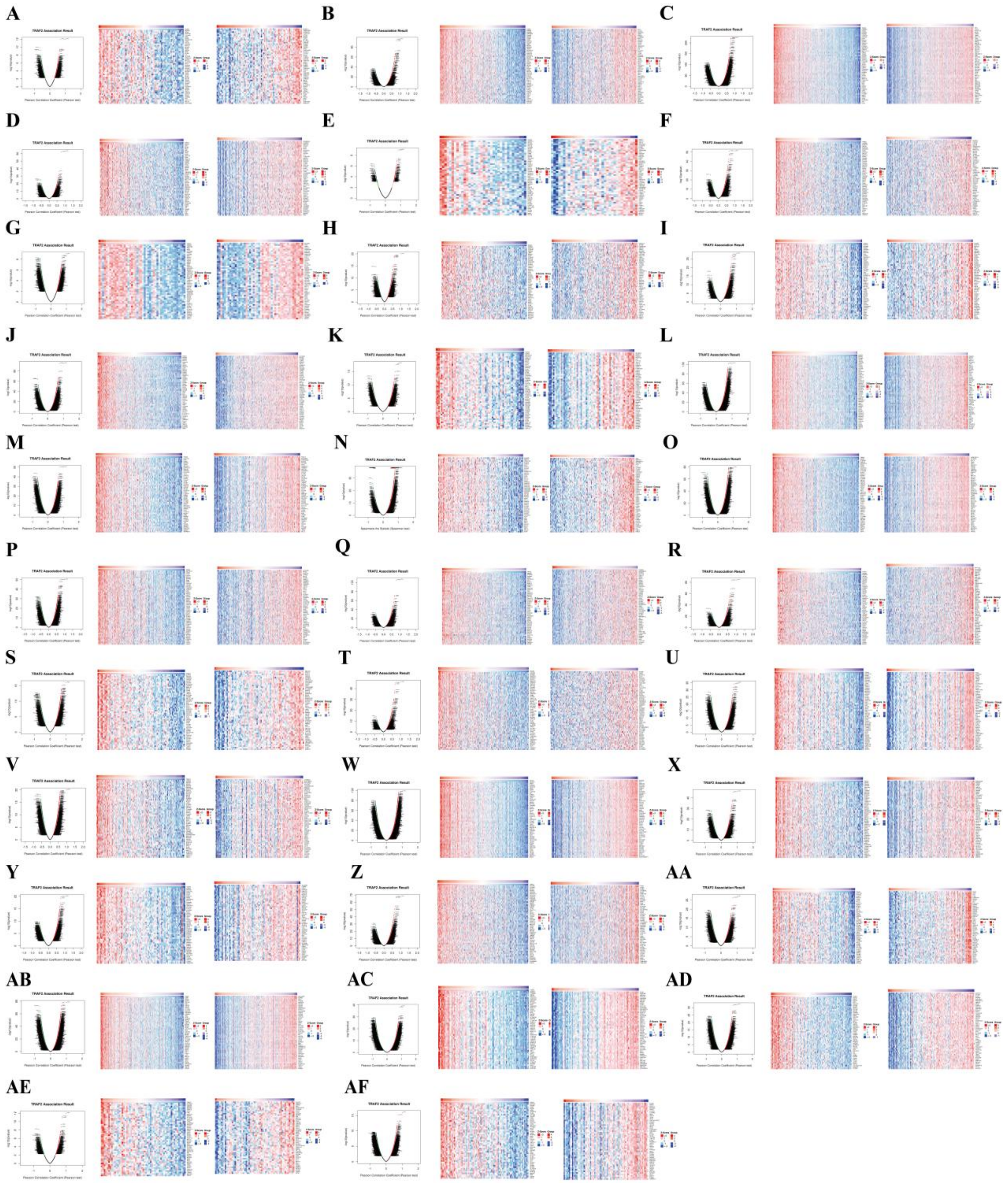

Fig.3 Heatmap illustrating the correlation between TRAF2 differentially expressed genes in cancer and up to 50 significant genes positively and negatively correlated with TRAF2.(A)ACC, (B)BLCA, (C)BRCA, (D)CESC, (E)CHOL, (F)COAD, (G)DLBC, (H)ESCA, (I)GBM, (J)HNSC, (K)KICH, (L)KIRC, (M)KIRP, (N)LAML, (O)LGG, (P)LIHC, (Q)LUAD, (R)LUSC, (S)MESO, (T)OV, (U)PAAD, (V)PCPG, (W)PRAD, (X)SARC, (Y)SKCM, (Z)STAD, (AA)TGCT, (AB)THCA, (AC)THYM, (AD)UCEC, (AE)UCS, (AF)UVM.

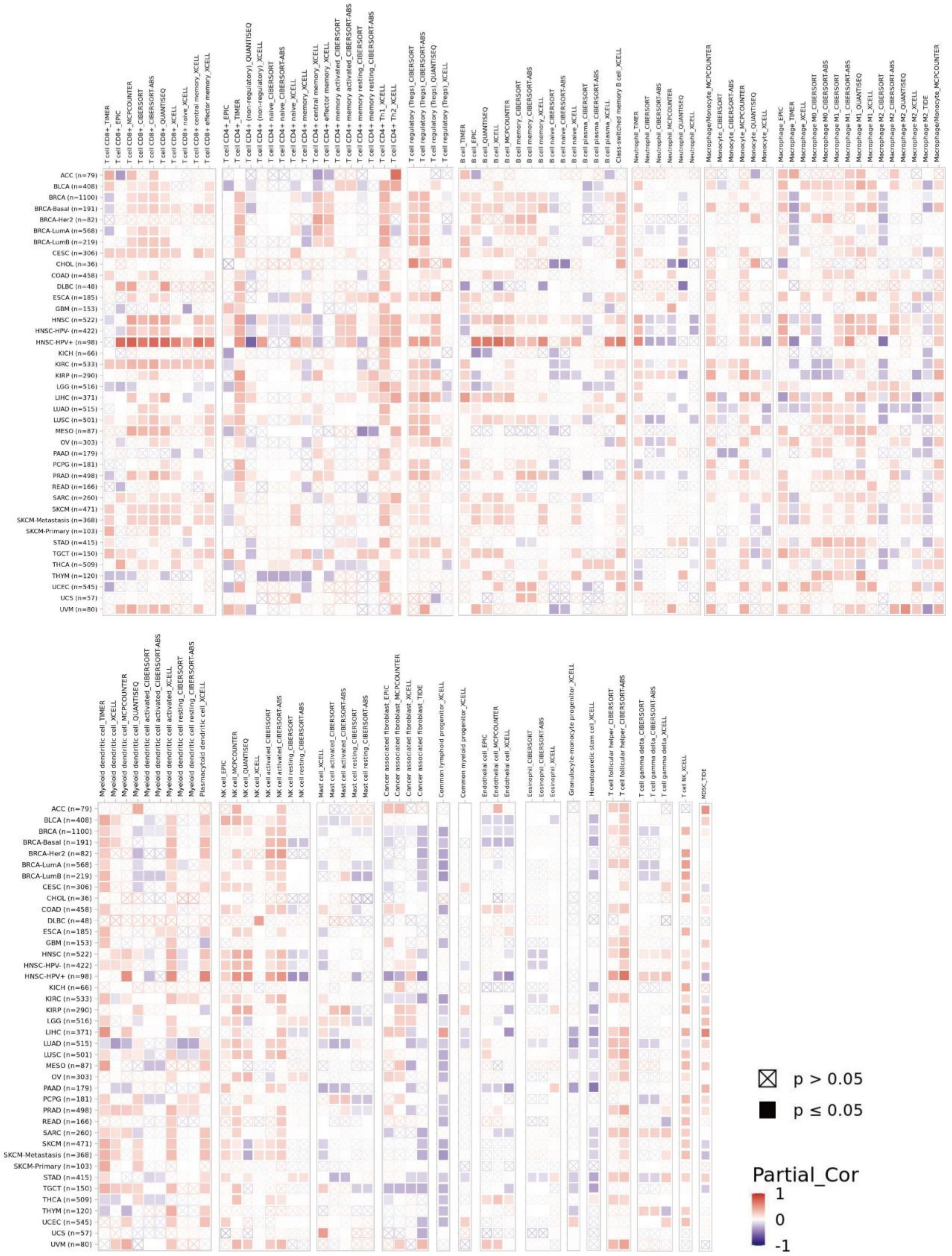

Fig.4 The relationship between TRAF2 and varying levels of immune cell infiltration was analyzed using the TIMER2.0 tools.

Supplementary Figure 5.

Fig.9 A

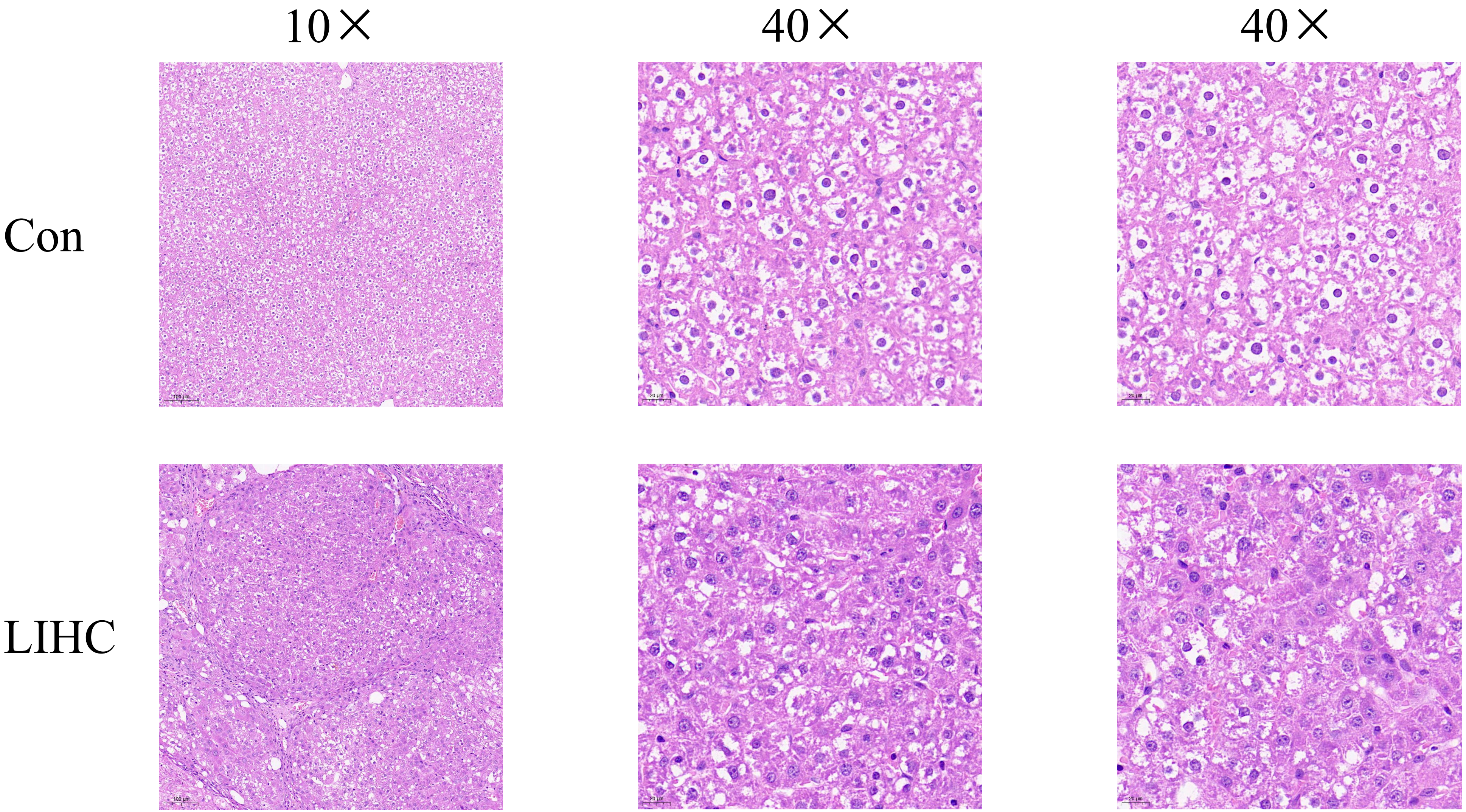

Supplementary Figure 5.

Fig.9 B

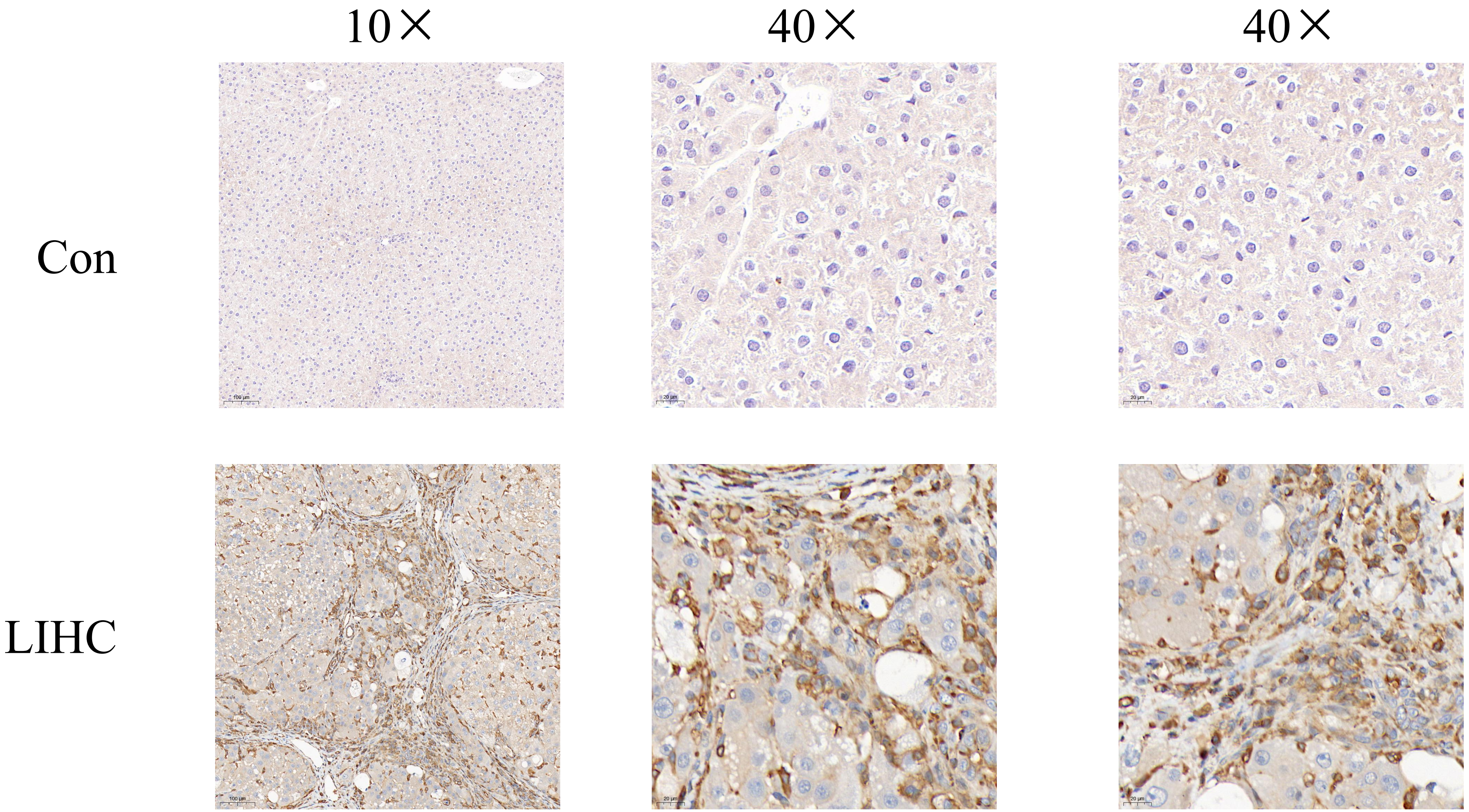

Supplementary Figure 5.

Fig.9 C

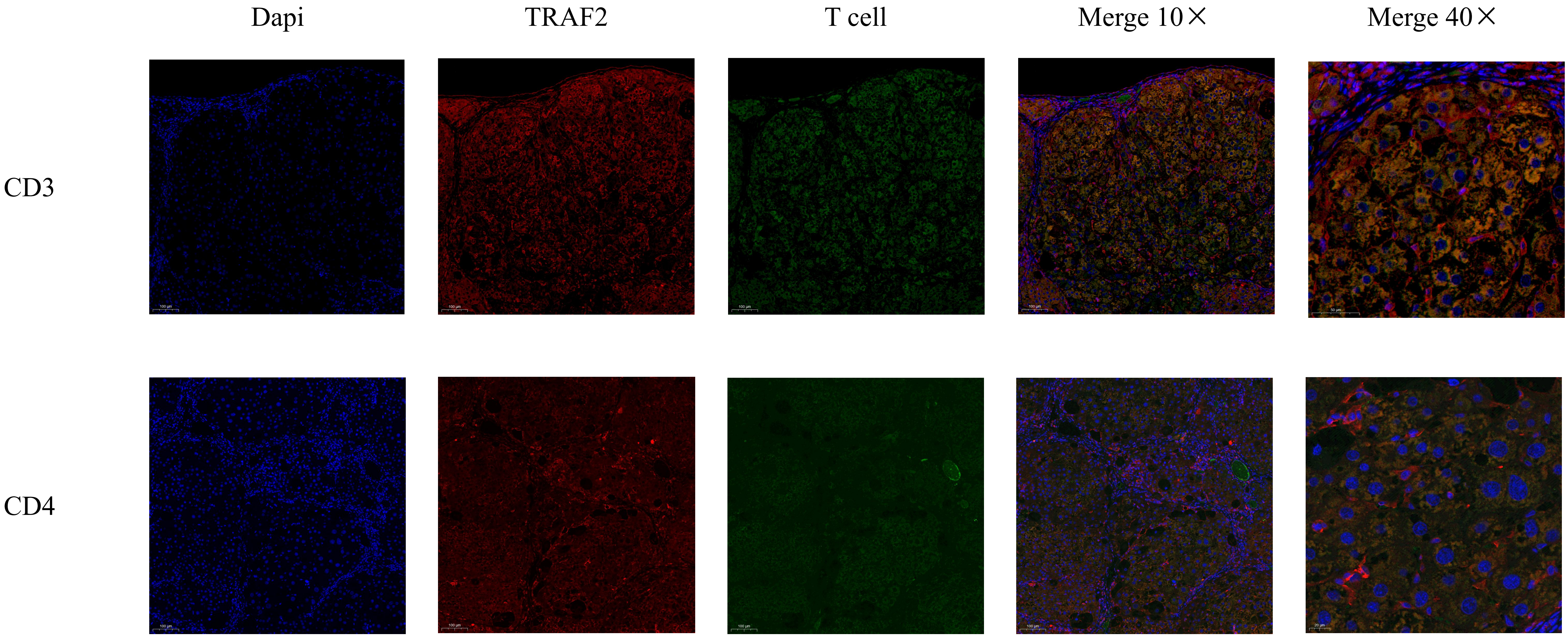

Supplementary Figure 5.

Fig.9 C

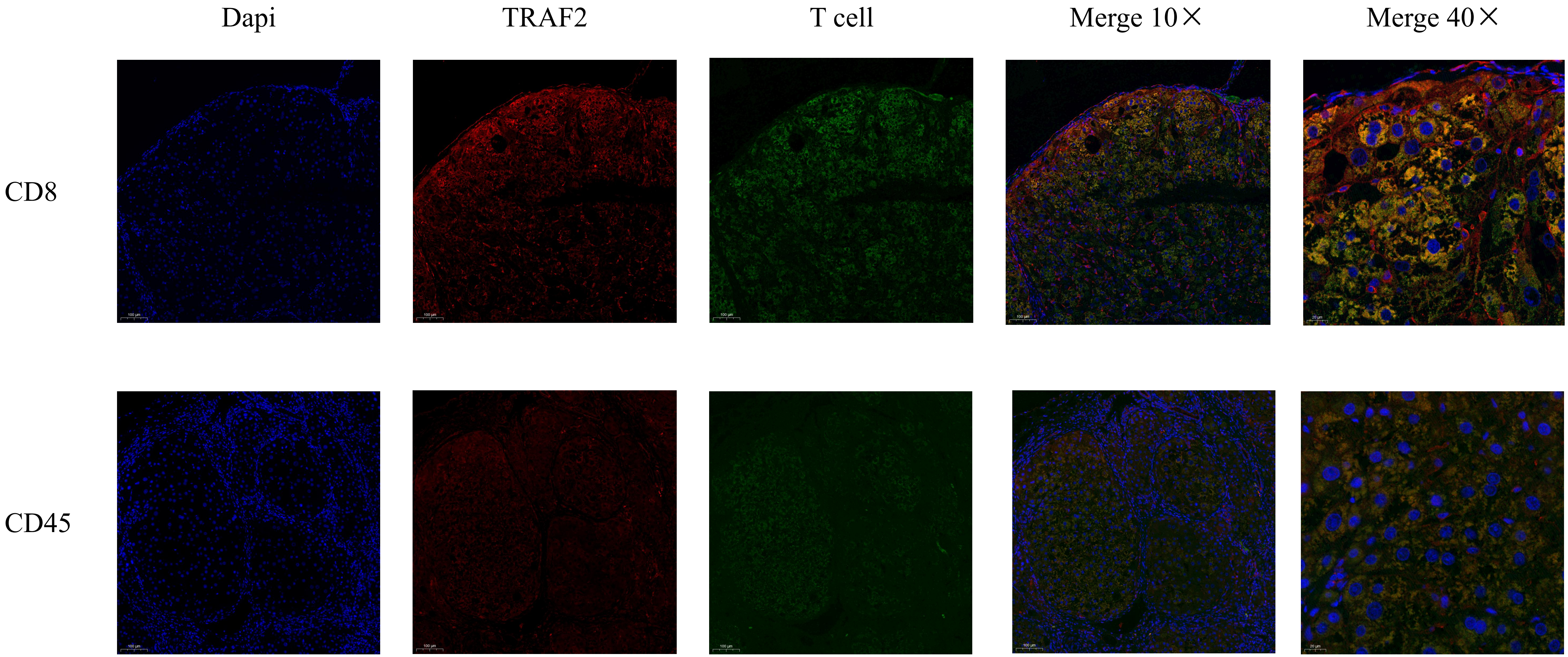

Supplementary Figure 6.

Fig.10 B

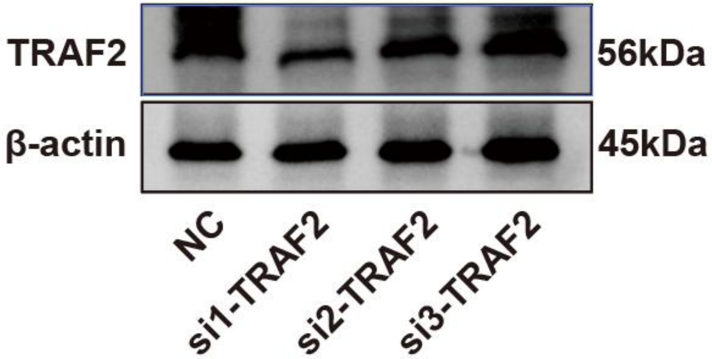

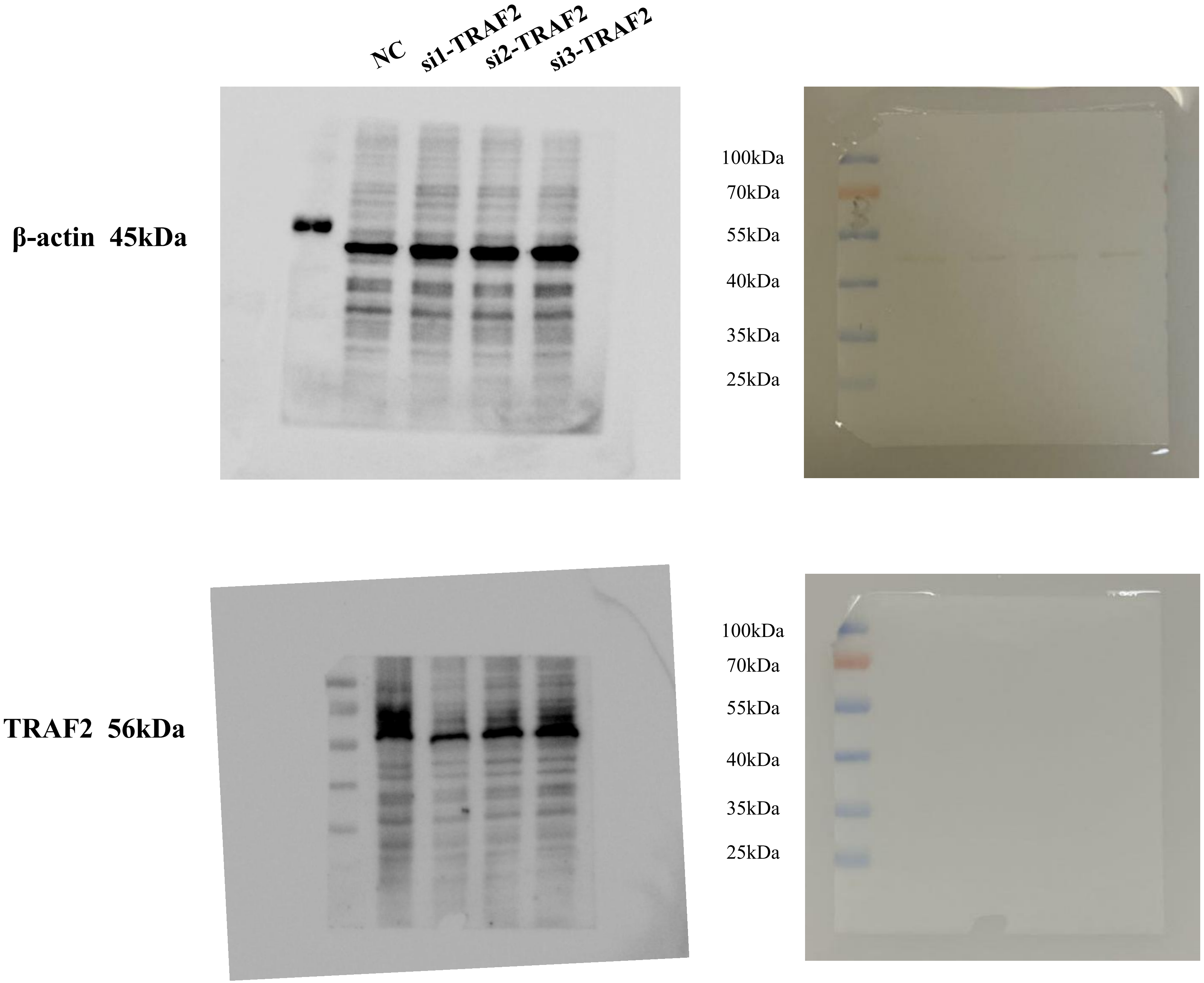

The red checkboxes are the bands presented in the manuscript image.

Supplementary Figure 6.

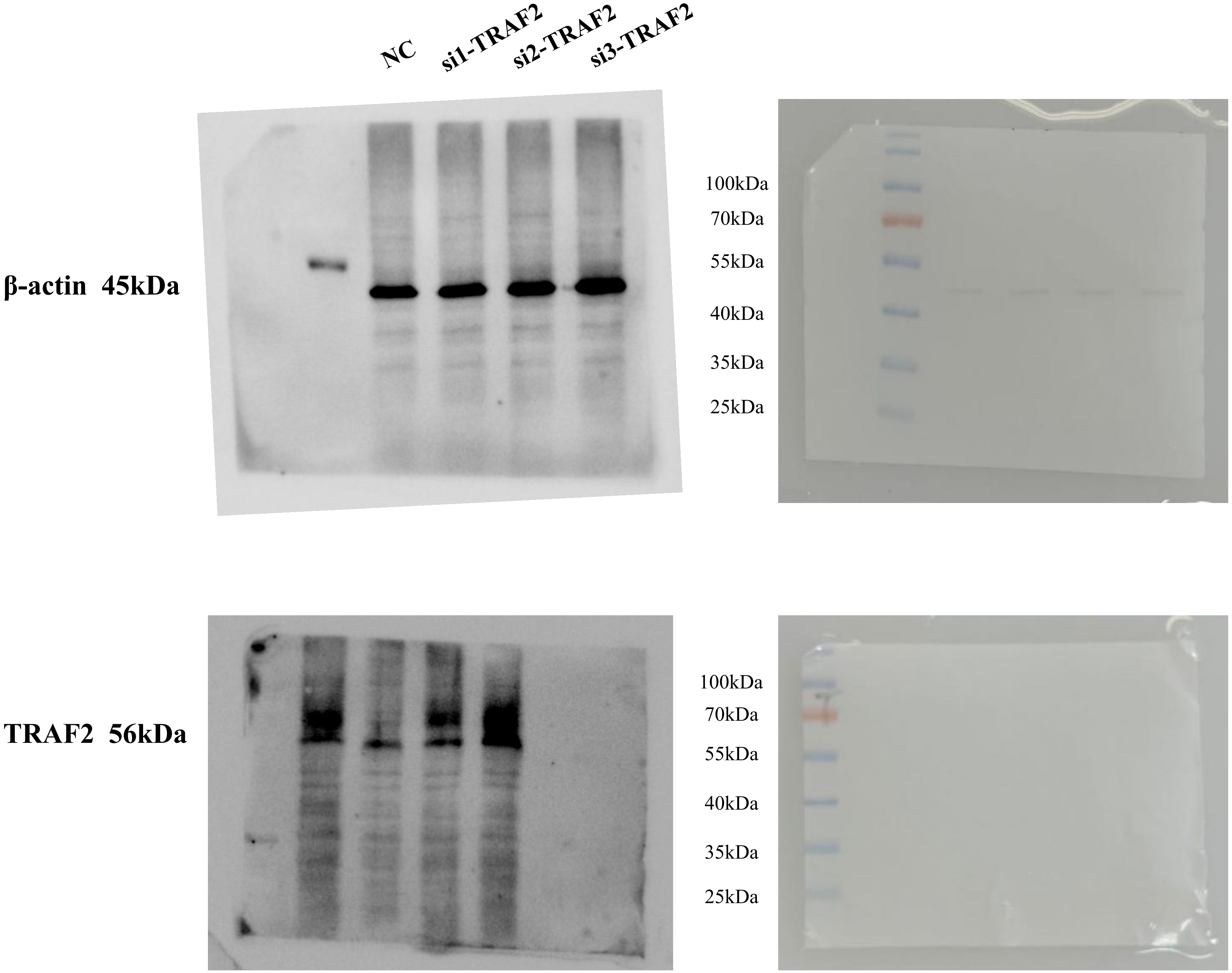

Supplementary Figure 6.

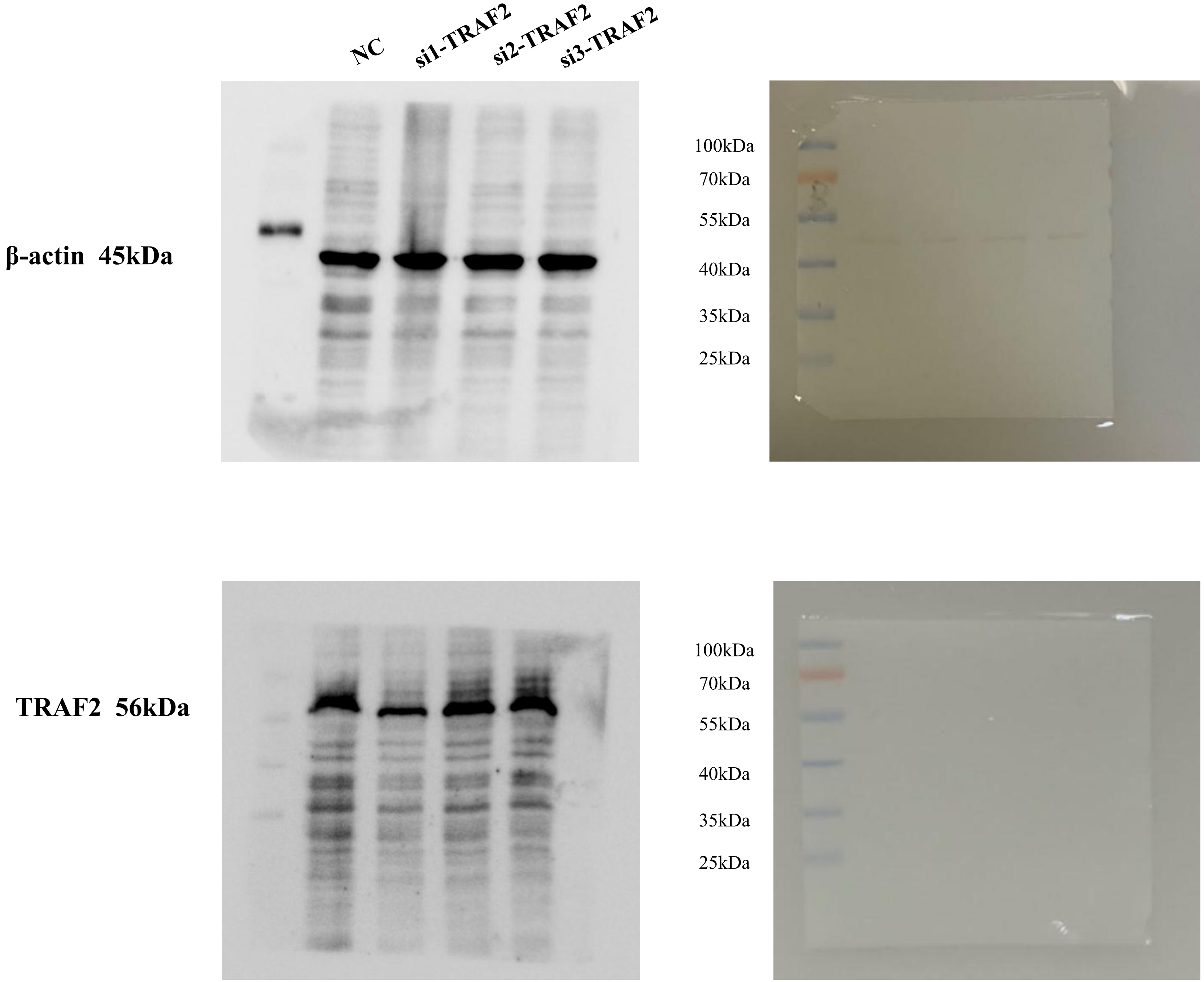

Supplementary Figure 7.

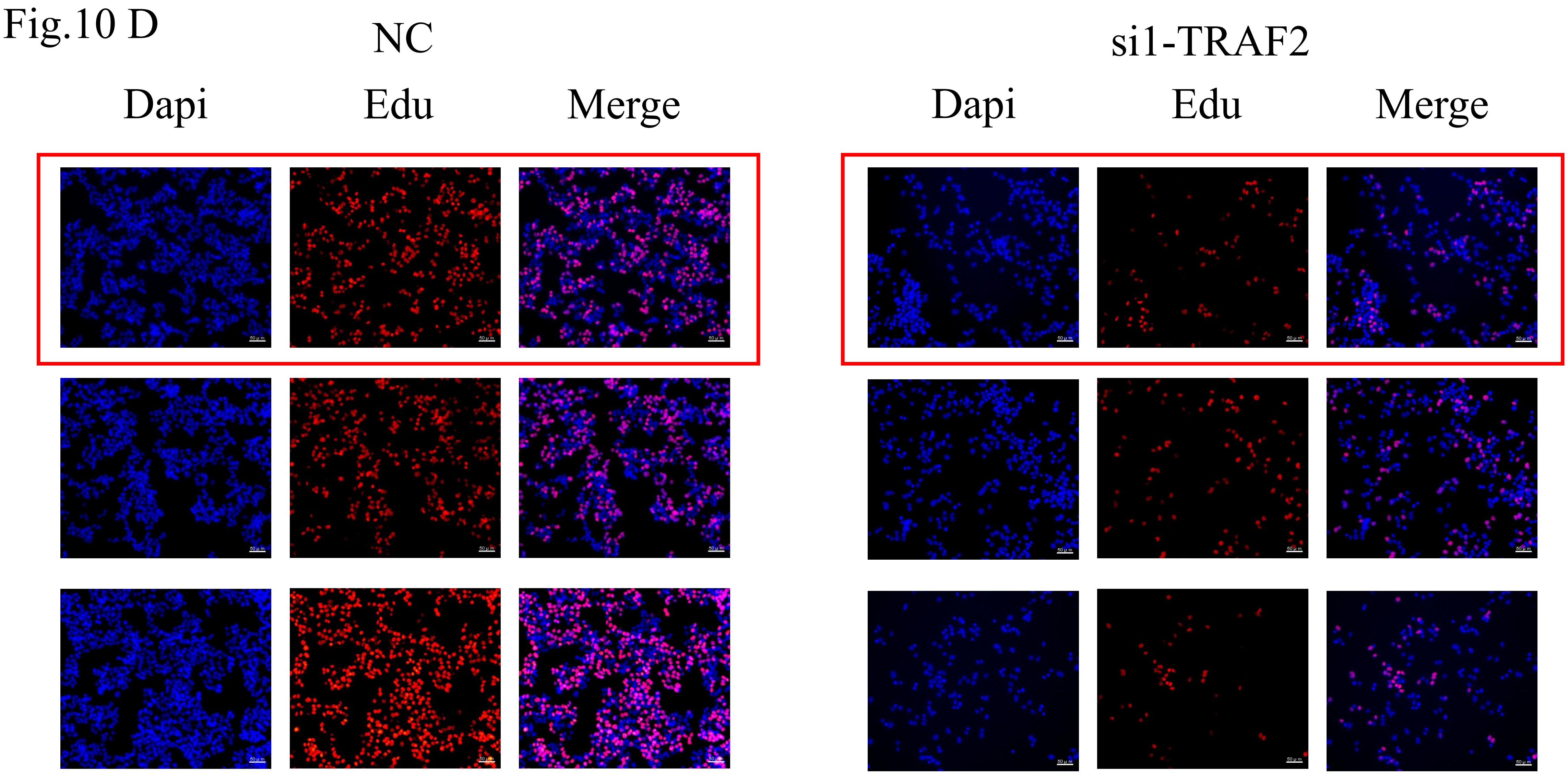

The red checkboxes are the bands presented in the manuscript image. Observed under a 20 × microscope.

Supplementary Figure 8.

Fig.10 F

NC

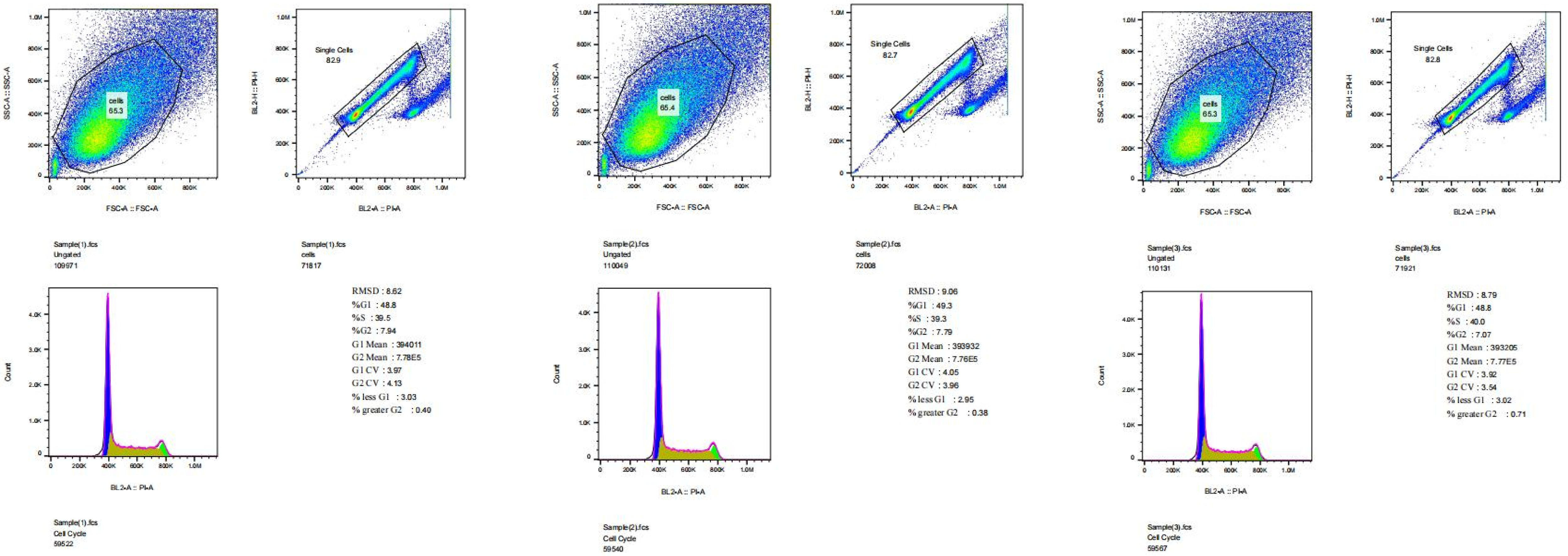

Supplementary Figure 8.

Fig.10 F

si1-TRAF2

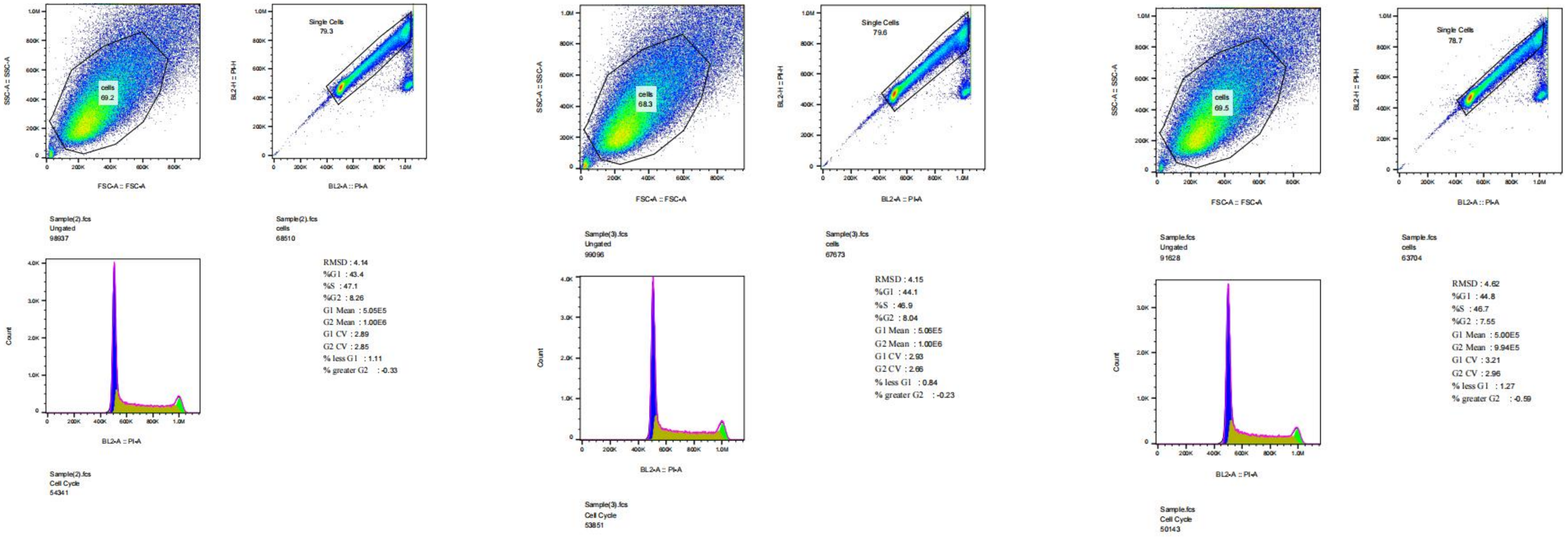

Supplementary Figure 9.

Fig.10 G

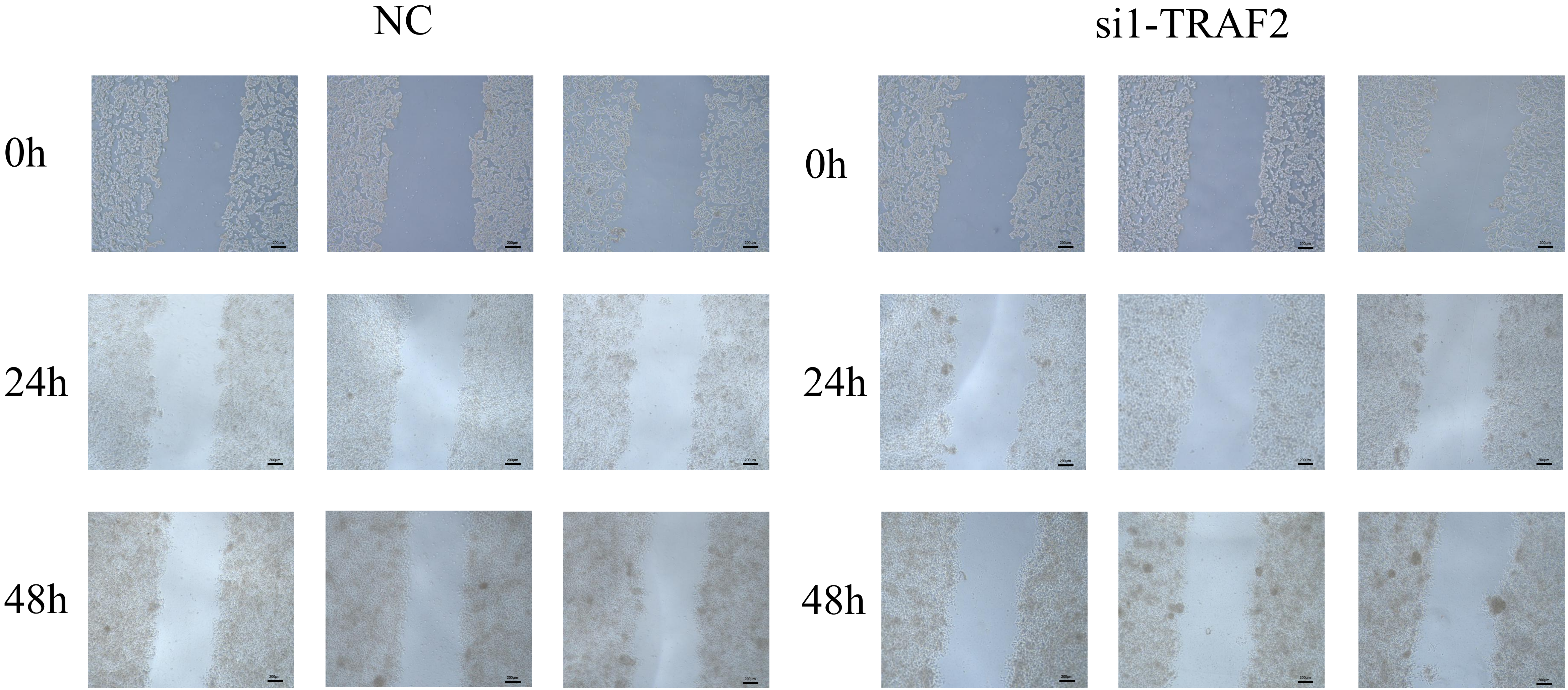

Observed under a 20 × microscope.

Supplementary Figure 10.

Fig.10 H

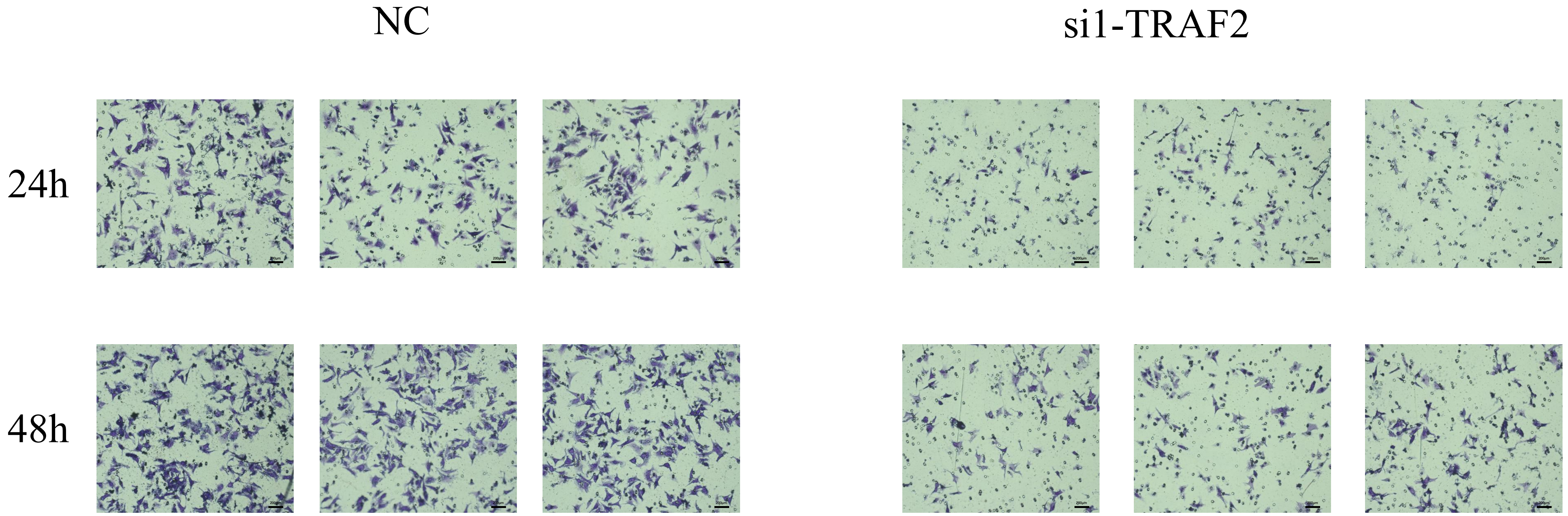

Observed under a 20 × microscope.
